# Molecular Population Genetics

**DOI:** 10.1534/genetics.116.196493

**Published:** 2017-03-03

**Authors:** Sònia Casillas, Antonio Barbadilla

**Affiliations:** * Institut de Biotecnologia i de Biomedicina, Universitat Autònoma de Barcelona, 08193, Spain; † Departament de Genètica i Microbiologia, Universitat Autònoma de Barcelona, 08193, Spain

**Keywords:** *Drosophila*, molecular population genetics, population genomics, neutral theory, distribution of fitness effects, genetic draft, linked selection, Hill–Robertson interference, population multi-omics, FlyBook

## Abstract

Molecular population genetics aims to explain genetic variation and molecular evolution from population genetics principles. The field was born 50 years ago with the first measures of genetic variation in allozyme loci, continued with the nucleotide sequencing era, and is currently in the era of population genomics. During this period, molecular population genetics has been revolutionized by progress in data acquisition and theoretical developments. The conceptual elegance of the neutral theory of molecular evolution or the footprint carved by natural selection on the patterns of genetic variation are two examples of the vast number of inspiring findings of population genetics research. Since the inception of the field, *Drosophila* has been the prominent model species: molecular variation in populations was first described in *Drosophila* and most of the population genetics hypotheses were tested in *Drosophila* species. In this review, we describe the main concepts, methods, and landmarks of molecular population genetics, using the *Drosophila* model as a reference. We describe the different genetic data sets made available by advances in molecular technologies, and the theoretical developments fostered by these data. Finally, we review the results and new insights provided by the population genomics approach, and conclude by enumerating challenges and new lines of inquiry posed by increasingly large population scale sequence data.

## 1966–2016: 50 Years of Molecular Population Genetics

HALF a century ago, two seminal articles inaugurated the field of molecular population genetics. Applying the technique of protein gel electrophoresis to several allozyme loci, the first measures of genetic variation in the species *Drosophila pseudoobscura* (Lewontin and Hubby 1966) and humans (Harris 1966) were provided. At this time, population genetics had built an extensive and sophisticated theoretical foundation; integrating principles of Mendelian inheritance with forces affecting changes in allele frequency in populations that sought to formalize the Darwinian view that biological evolution is a population process by which genetic variation within species is transformed into genetic variation between species (Mayr 1963). But because of the technical inability to measure genetic variation for all but a few loci, this exhaustive formal exercise occurred in a virtual factual vacuum. With almost no data, models were totally general; unrestricted by the contingent world (Lewontin 1974). After decades of struggling to measuring genetic variation, copious data on electrophoretic variation initiated at last the necessary dialog between data and theory. Since then, this dialog has continued to catalyze the main advances in the field.

How far are we today, 50 years later? The genomic revolution has generated detailed population genetic data, far exceeding the dreams of any premolecular population geneticist. Big data samples of complete genome sequences of many individuals from natural populations of many species have transformed population genetics inferences on samples of loci to population genomics: the analysis of genome-wide patterns of DNA variation within and between species. Catalogs of nearly all polymorphic variants are currently available for model species such as *D. melanogaster* (Langley *et al.* 2012; Mackay *et al.* 2012; Huang *et al.* 2014; Grenier *et al.* 2015; Lack *et al.* 2015), yeasts (Liti *et al.* 2009; Strope *et al.* 2015), *Arabidopsis thaliana* (Cao *et al.* 2011; Gan *et al.* 2011; 1001 Genomes Consortium 2016), *Caenorhabditis elegans* (Andersen *et al.* 2012), as well as humans (Durbin *et al.* 2010; 1000 Genomes Project Consortium 2012, 2015; Sudmant *et al.* 2015). In the coming years, population genomic data will continue to grow in both amount of sequences and number of species (Ellegren 2014; Tyler-Smith *et al.* 2015). The current human single nucleotide polymorphism (SNP) database lists 100,815,862 validated SNPs (dbSNP, April 2016; https://www.ncbi.nlm.nih.gov/SNP/). In *D. melanogaster*, >6,000,000 natural variants (SNPs and indels) have been described (Huang *et al.* 2014) to date. What is the power of these millions of segregating variants in the genomes of species to solve the field’s great obsession (Gillespie 1991) about the evolutionary forces causing the observed patterns of genetic variation? Is this vast information all we need to explain molecular evolution?

In his influential book, *The Genetic Basis of Evolutionary Change*, Lewontin (1974) assesses the first impact of electrophoretic variation data on the body of theory developed previously. He wonders if the population genetics machinery is empirically insufficient, no more because of lack of data, but because of an incompleteness in the theoretical parameters that made it incapable of accounting for the observations. The advances in molecular evolutionary genetics have subsequently enriched the field with many new concepts, terms, processes, molecular techniques, and statistical and computational methods. But remarkably, the fundamental forces of evolution established by the founding fathers of the field (Fisher 1930; Wright 1931; Haldane 1932; Kimura 1955), namely natural selection, genetic drift, mutation, recombination, and gene flux, are still the essential explanatory factors used for understanding the population genetic basis of evolutionary change (Lynch 2007; Charlesworth 2010).

In the next pages, we focus largely on what we have learned about the intragenomic component of genetic variation; showing that genome variation at a given genomic region depends not only on the sequence functional class (synonymous, nonsynonymous, intron, *etc*.) but also on the underlying genomic context such as level of recombination or mutation rate, gene density, chromosomal region, or chromosome associated with such a region. We first describe the main landmarks along the 50 years of molecular population genetics. For clarity, we consider separately advances in data acquisition and theory development. We describe the different genetic data sets that the successive molecular technologies have made available, and then the theoretical contributions and improvements fostered by the data. The relevance of *Drosophila* in this journey will be emphasized. Finally, we review the results and new insights provided by the population genomics approach, followed by the enumeration of challenges and new lines of inquiry posed by the present population genomics (multi-omics) momentum.

## Drosophila as a Model Organism for Population Genetics

First introduced as a research tool in the early 20th century (Morgan *et al.* 1915; Muller 1927), *Drosophila* has played a crucial role in all fields of genetic analysis, including ecology, speciation, development, and also population genetics (Powell 1997). Following early studies of chromosomal inversion polymorphisms (Dobhansky 1937; Dobzhansky and Sturtevant 1938), Drosophilists pioneered the initial surveys of molecular genetic variation (see next section) and *Drosophila* was used extensively to study the forces shaping genetic variation in natural populations (Ayala *et al.* 1974; Singh and Rhomberg 1987).

**Figure 1:**
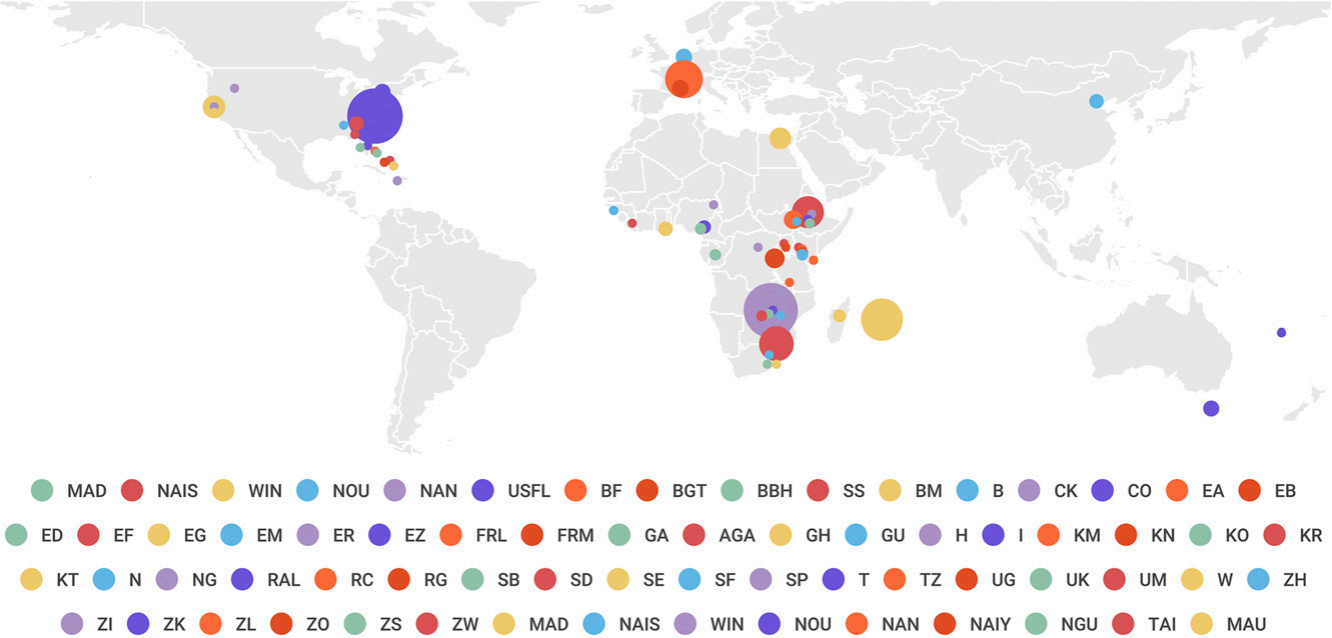
Population genomics resources available for four *Drosophila* species. ● represents sequenced populations, and the size of the ● is proportional to the number of individuals sequenced. See an interactive and updateable version of this figure with additional information about each population at http://flybook-mpg.uab.cat. *D. melanogaster populations:* USTB, Tampa Bay, FL, *n* = 2; UST, Thomasville, GA, *n* = 2; USS, Selva, AL, *n* = 2; USB, Birmingham, AL, *n* = 2; USM, Meridian, MS, *n* = 2; USFL, Sebastian, FL, *n* = 2; BF, Freeport, Bahamas, *n* = 2; BGT, George Town, Bahamas, *n* = 2; BBH, Bullocks Harbor, Bahamas, *n* = 2; SS, Cockburn Town, San Salvador, n = 2; BM, Mayaguana, Bahamas, *n* = 2; B, Beijing, China, *n* = 15; CK, Kisangani, Congo, *n* = 2; CO, Oku, Cameroon, *n* = 13; EA, Gambella, Ethiopia, *n* = 24; EB, Bonga, Ethiopia, *n* = 5; ED, Dodola, Ethiopia, *n* = 8; EF, Fiche, Ethiopia, *n* = 69; EG, Cairo, Egypt, *n* = 32; EM, Masha, Ethiopia, *n* = 3; ER, Debre Birhan, Ethiopia, *n* = 5; EZ, Ziway, Ethiopia, *n* = 5; FRL, Lyon, France, *n* = 96; FRM, Montpellier, France, *n* = 20; GA, Franceville, Gabon, *n* = 10; AGA, Athens, GA, *n* = 15; GH, Accra, Ghana, *n* = 15; GU, Dondé, Guinea, *n* = 7; H, Port Au Prince, Haiti, *n* = 2; I, Ithaca, NY, *n* = 19; KM, Malindi, Kenya, *n* = 4; KN, Nyahururu, Kenya, *n* = 6; KO, Molo, Kenya, *n* = 4; KR, Marigat, Kenya, *n* = 6; KT, Thika, Kenya, *n* = 2; N, Houten, Netherlands, *n* = 19; NG, Maiduguri, Nigeria, *n* = 6; RAL, *n* = 205; RC, Cyangugu, Rwanda, *n* = 2; RG, Gikongoro, Rwanda, *n* = 27; SB, Barkly East, South Africa, *n* = 5; SD, Dullstroom, South Africa, *n* = 81; SE, Port Edward, South Africa, *n* = 3; SF, Fouriesburg, South Africa, *n* = 5; SP, Phalaborwa, South Africa, *n* = 37; T, Sorell, Tasmania, Australia, *n* = 18; TZ, Uyole, Tanzania, *n* = 3; UG, Namulonge, Uganda, *n* = 6; UK, Kisoro, Uganda, *n* = 5; UM, Masindi, Uganda, *n* = 3; W, Winters, CA, *n* = 35; ZH, Harare, Zimbabwe, *n* = 4; ZI, Siavonga, Zambia, *n* = 197; ZK, Lake Kariba, Zimbabwe, *n* = 3; ZL, Livingstone, Zambia, *n* = 1; ZO, Solwezi, Zambia, *n* = 2; ZS, Sengwa, Zimbabwe, *n* = 5; ZW, Victoria Falls, Zimbabwe, *n* = 9; MAD, Tampa Bay, FL, *n* = 2; NAIS, Thomasville, GA, *n* = 2; WIN, Selva AL, *n* = 2; NOU, Birmingham, AL, *n* = 2; NAN, Meridian, MS, *n* = 2. *D. simulans* populations: MAD, Madagascar, *n* = 12; NAIS, Nairobi, Kenya, *n* = 10; WIN, Winters, CA, *n* = 2; NOU, Noumea, New Caledonia, *n* = 1; NAN, Nanyuki, Kenya, *n* = 1. *D. yakuba* populations: NAIY, Nairobi, Kenya, *n* = 10; NGU, Nguti, Cameroon, *n* = 10; TAI, Taï Rainforest, Liberia, *n* = 1. *D. mauritiana* populations: MAU, Mauritius, *n* = 117.

As the third eukaryote and the second metazoan to be fully sequenced, *D. melanogaster* was chosen to explore the application of complete genome sequencing by whole-genome shotgun in eukaryotic genomes (Rubin 1996; Adams *et al.* 2000). More recently, the development of high-throughput sequencing technologies allowed the sequencing of >200 complete genomes of *D. melanogaster* from a population sampled in Raleigh (RAL), NC [*Drosophila* Genetic Reference Panel (DGRP)] (Mackay *et al.* 2012; Huang *et al.* 2014). Following this study, 100s of individuals from many other populations were sequenced [*Drosophila* Population Genomics Project (DPGP); Global diversity lines] (Langley *et al.* 2012; Grenier *et al.* 2015; Lack *et al.* 2015) and today >1000 complete genomes are available for *D. melanogaster* (Lack *et al.* 2015, 2016) (Figure 1). In addition, several other *Drosophila* species have been completely sequenced and used for comparative genomic studies (Drosophila 12 Genomes Consortium *et al.* 2007; Hales *et al.* 2015). Population genomic resources are available for 27 lines of *D. simulans* (Begun *et al.* 2007; Rogers *et al.* 2014), 21 lines of *D. yakuba* (Begun *et al.* 2007; Rogers *et al.* 2014), and 117 pooled samples of *D. mauritiana* (Nolte *et al.* 2013; Garrigan *et al.* 2014) (Figure 1). The availability of these sequence data provides the fly lineage with a unique resource on which to test the molecular population genetics hypotheses and eventually understand the evolutionary dynamics of genetic variation in populations.

## The Data: From Empirical Insufficiency to the Present Flood of Genome Variation

A primary concept of the modern evolutionary synthesis period (1930s–1960s) was the primary role of natural selection to explain evolution (Mayr and Provine 1980), while largely ignoring effects of genetic drift. Two different views emerged. The so-called *classical hypothesis* supported the role of natural selection in purging the population of most genetic variation, predicting that most loci are homozygous for the wild-type allele (Muller and Kaplan 1966). The *balance hypothesis* postulated that natural selection actively maintained high levels of genetic diversity in populations, and that a large proportion of loci are therefore polymorphic (Dobzhansky 1970; Ford 1971). Note that under the second hypothesis, evolution in the face of fluctuations in environmental conditions over time may be rapid since selection can act on existing variants; while under the first hypothesis evolution may be constrained by the availability of new advantageous mutations.

Resolving the controversy of how much variation within a natural population there is at an average locus required large studies to empirically measure genetic diversity in populations. This was made possible for the first time in 1966 with the start of the allozyme era (Lewontin and Hubby 1966; reviewed by Charlesworth *et al.* 2016). Later on, allozymes were replaced by a much more informative source of genetic variation data that came from the sequencing of nucleotide sequences (Kreitman 1983), and eventually by the sequencing of complete genomes (Begun *et al.* 2007; Langley *et al.* 2012; Mackay *et al.* 2012). In this section we describe these three stages to survey molecular genetic variation during the last 50 years, which range from the empirical insufficiency of allozymes to the present flood of genome variation data.

### The allozyme era: setting the stage for the neutralist–selectionist debate

Population genetics entered the molecular age with the publication of seminal articles describing electrophoretically detectable variation—or allozymes (*i.e.*, proteins differing in electrophoretic mobility as a result of allelic differences in the protein sequence, which ultimately result from the existence of variation in the corresponding DNA sequence)—in *D. pseudoobscura* (Lewontin and Hubby 1966) and also in humans (Harris 1966). A few dozen different soluble proteins were studied in 100s of species, mostly enzymes with well-understood metabolic roles. Genetic diversity was measured in two ways: the average proportion of loci that are heterozygous in an individual [*heterozygosity* or *gene diversity* (*H*)], and the average proportion of loci that are polymorphic in the population [*gene polymorphism* (*P*)]. The results of such electrophoretic surveys revealed a large amount of genetic variation in most populations (Lewontin 1974, 1985), much more than had been predicted, and seemed to unequivocally support the balance rather than the classical hypothesis. Specifically, 43% of loci were found to be polymorphic in *Drosophila*, and *H* is ∼12%. Furthermore, levels of genetic diversity were found to vary nonrandomly among populations, species, higher taxa, and several ecological, demographic, and life-history parameters (Nevo *et al.* 1984). For example, most invertebrates (including *Drosophila*) appear to be highly polymorphic; whereas reptiles, birds, and mammals are only about half as variable on average (*e.g.*, in humans, *P* and *H* are about 28 and 7%, respectively), and fish and amphibians are intermediate in their variability. These data showed that population size is a key parameter in population genetics and the neutral theory was derived to account for molecular evolution [Box 1; see *The* (*nearly*) *neutral theory as the paradigm*], setting the stage for the long-lasting neutralist *vs.* selectionist debate. While large populations are expected to accumulate more variation, the small differences in the levels of genetic diversity seen among distant species were not sufficient to explain the large differences in their population sizes (Lewontin 1974). In particular, even though the total range in population sizes over all species exceeds 20 orders of magnitude (Lynch 2006), allozyme diversity varies by less than a power of 4 (Bazin *et al.* 2006), an observation which is often known as Lewontin’s paradox (Lewontin 1974).

Box 1. **Implications of Kimura’s Neutral Theory**

**Figure 2:**
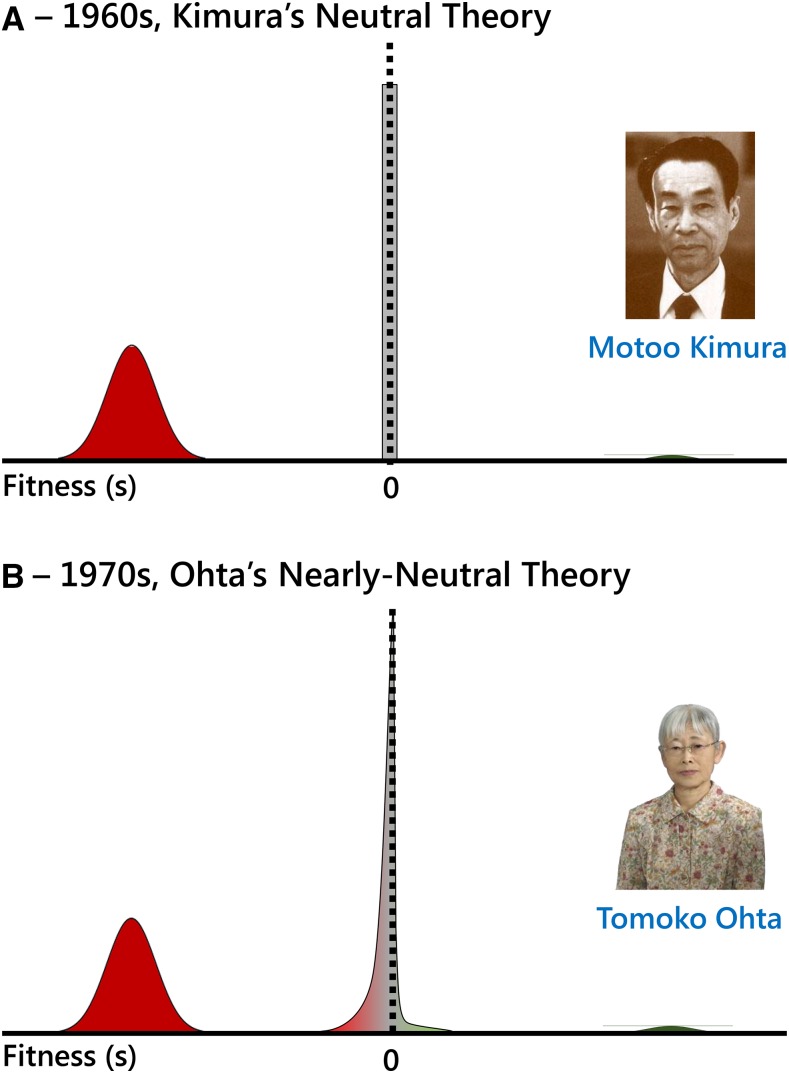
DFE according to the (nearly) neutral theory of molecular evolution. (A) In the 1960s, according to the Kimura’s neutral theory. (B) In the 1970s, after the extension of the neutral theory by Ohta. Different selection coefficients of mutations are colored in a gradient from maroon (strongly deleterious), red (slightly deleterious), gray (neutral), light green (slightly advantageous), and dark green (advantageous).

**Figure 3:**
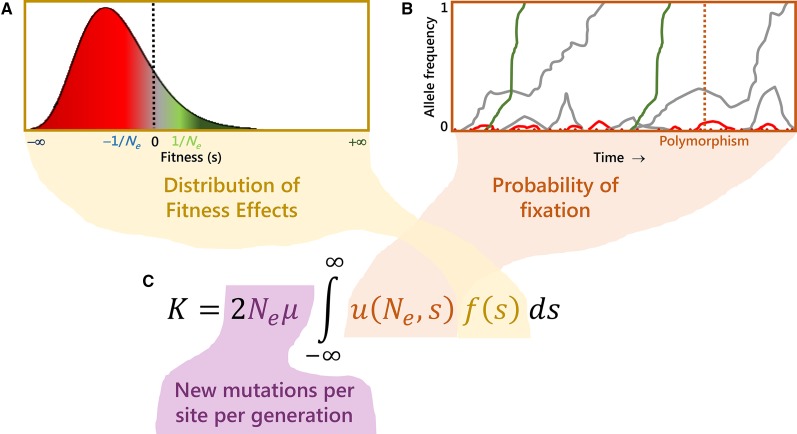
Molecular evolutionary rate (*K*) as a function of (A) the DFE, (B) the probability of fixation of new mutations entering the population, and (C) the rate at which new mutations enter the population per site per generation (see text for details). Different selection coefficients of mutations are colored in a gradient from maroon (strongly deleterious), red (slightly deleterious), gray (neutral), light green (slightly advantageous), and dark green (advantageous).
Mutations that have fitness effects on the order of 1/*N*_e_ are nearly neutral [slightly deleterious if the selection coefficient *s* is negative (Figure 3A, red), or slightly advantageous when *s* is positive (Figure 3A, light green)], they have small effects on fitness, and their fate hinges on a combination of natural selection and genetic drift.
Mutations with fitness effects >1/10*N*_e_ are strongly deleterious (if *s* is negative; Figure 3A, maroon) or strongly advantageous (if *s* is positive; Figure 3A, dark green), and their fates are mainly determined by natural selection.

In the late 1960s, Motoo Kimura suggested that patterns of protein polymorphism seen in nature were consistent with the view that most polymorphisms and fixed differences between species are either strongly deleterious or selectively neutral (Figure 2A). This proposal was called the neutral theory of molecular evolution (Kimura 1968) (also known as the mutation-drift balance hypothesis) with the following principal assertions (Kimura 1968, 1983):

1. Strongly deleterious mutations are rapidly removed from the population (Figure 3B, small maroon dots close to the *x*-axis), and adaptive mutations are rapidly fixed (Figure 3B, green); therefore, most variation within species (Figure 3B, dotted vertical line) is the result of neutral mutations (Figure 3B, gray).
2. Polymorphisms are transient (on their way to loss or fixation) rather than balanced by selection.
3. The level of polymorphism in a population (θ) is a function of the neutral mutation rate (μ_0_) and the effective population size (*N*_e_): θ *=* 4*N*_e_μ_0_ (in diploids). Larger populations are expected to have a higher heterozygosity, as reflected in the greater number of alleles segregating at a time.
4. A steady-state rate at which neutral mutations are fixed in a population (*K*) equals the neutral mutation rate: *K* = μ_0_. Therefore, the average time between consecutive neutral substitutions is independent of population size (1*/*μ_0_).

**Figure:**
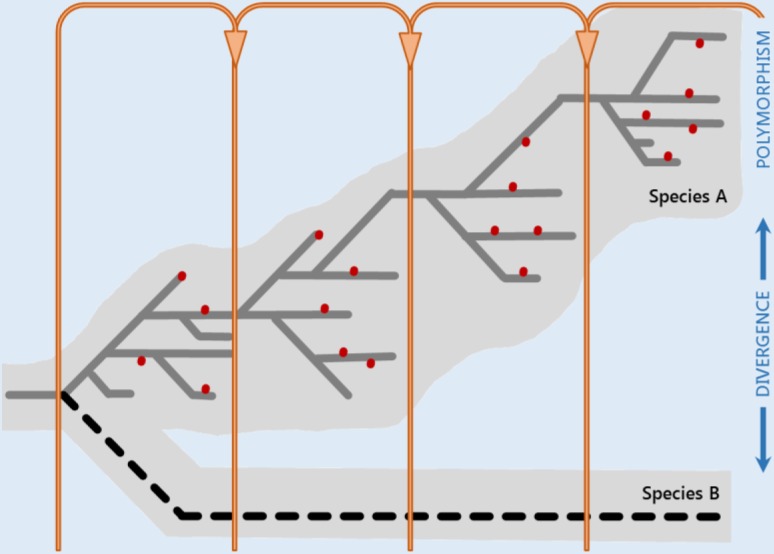
**Kimura’s neutral theory of molecular evolution**. By postulating the revolutionary new concept of neutral variants, Kimura’s neutral theory summarizes molecular evolution in one the most elegant mathematical expressions in science. The expression $K=\mu_{0}$ (the rate of molecular evolution equals the neutral mutation rate) unifies the three levels of genetic variation from its origin to its substitution in the population: mutation (individual level), polymorphism (population level), and divergence (species level). According to the neutral theory, intrapopulation polymorphism is just a random walk of variants in their process to fixation or loss (represented for species A: gray, neutral mutations; maroon, strongly deleterious mutations; see also Figure 3B). Orange arrows represent the average lifetime of a neutral mutation from its appearance to its fixation in the population (1/μ_0_).

While protein electrophoresis was extensively used to perform large-scale surveys of genetic diversity in a wide range of species (Nevo *et al.* 1984), the limitations of the method were well known. First, allozyme polymorphisms can only be observed for DNA variation that alters the amino acid sequence. Second, only those amino acid changes that affect the mobility of a protein in a gel (mostly associated with charge changes) can be detected by electrophoresis, and these represent only about one-fourth of all possible mutational changes that lead to an amino acid substitution (Lewontin 1991). Ohta and Kimura (1973) proposed the charge-state model (or stepwise mutation model) to explain the results of electrophoretic studies while accommodating these limitations of allozyme markers, and this model was further followed by some extensions (Brown *et al.* 1981). However, Barbadilla *et al.* (1996) showed that if charge is considered synonymous with electrophoretic mobility, as in the charge-state model, then we expect, for almost any given scenario, a symmetrical bell-shaped distribution of mobilities where charge classes with the highest frequency have an intermediate mobility. They conclude that the commonly observed frequency pattern of electrophoretic variants is purely a consequence of statistical relations and conveys no information about the underlying evolutionary forces. Also, they show that the discriminatory power of electrophoresis to detect protein variation is a decreasing function of the number of segregating sites. In summary, and given the limitations of protein electrophoresis to measure genetic variation, Lewontin (1991) assesses this initial stage in the analysis of genetic diversity not only as a *milestone* of evolutionary genetics, representing the initial stage in a journey to survey genetic variation in the populations; but also as a frustrating *millstone* because the boom of electrophoresis swamped the previous diversity of empirical work in evolutionary genetics, and because of the lack of fit of empirical data to the evolutionary genetics theory. It was apparent, then, that the direct study of DNA variation would be necessary to answer the questions that population genetics had already posed. In the words of Lewontin (1991): “Those of us who now study DNA sequence variation believe that at this level we will resolve the problems generated by electrophoretic studies and that finally, because the structure of the observation of DNA sequences is qualitatively different from observations of amino acid variation, that the ambiguities will disappear.”

### The nucleotide sequence era

Before the invention of PCR amplification and automated Sanger sequencing, the first surveys of DNA sequence variation were done in the 1980s using restriction enzymes to detect variation at restriction sites; an approach that was extensively used in *Drosophila* (Langley *et al.* 1982, 1988; Aquadro *et al.* 1986; Langley and Aquadro 1987; Schaeffer *et al.* 1988; Miyashita and Langley 1988; Aguadé *et al.* 1989b, 1992; Stephan and Langley 1989). A large number of phylogeographic studies were published, often analyzing one or several mitochondrial DNA (mtDNA) loci (Avise *et al.* 1987). Restriction mapping was the starting point for the development of new summary statistics to represent genetic diversity on DNA sequences, including the *nucleotide site diversity* (π), the equivalent of *H* for nucleotide sites (Nei and Li 1979). Furthermore, studies in *Drosophila* uncovered an intriguing pattern: regions of the genome with low recombination have very low levels of genetic variability (Aguadé *et al.* 1989a; Stephan and Langley 1989; Berry *et al.* 1991; Begun and Aquadro 1992; Martin-Campos *et al.* 1992; Stephan and Mitchell 1992; Langley *et al.* 1993). Begun and Aquadro (1992) published a landmark study reporting one of the most far-reaching observations in molecular evolution: the local rate of recombination is strongly positively correlated to the level of genetic variation. A mechanistic relationship between recombination and mutation seemed an obvious explanation. If recombination is indeed mutagenic, then regions of low recombination should also have a low mutation rate, and hence lower interspecific divergence according to the neutral theory (*K* = μ_0_, see below). However, levels of divergence were shown to be independent of local recombination rates, and thus the correlation between recombination rate and levels of polymorphism was attributed to the fixation of advantageous mutations and the associated hitchhiking effect. The lower the recombination of a region, the larger the hitchhiking effect, and thus the reduction of linked neutral variation; accounting for the observed correlation. This hitchhiking hypothesis seriously jeopardized the Kimura’s neutral theory of molecular evolution (see *Genetic draft as a selectionist alternative to the neutral theory* and *Recombination and linked selection*).

The first study of nucleotide sequence variation, by sequencing multiple copies of a complete contiguous region of the genome (a procedure known as resequencing), was conducted by Kreitman (1983) in the *Adh* gene region from 11 independently isolated chromosomes of five natural populations of *D. melanogaster*. This pioneering study used the very laborious manual Maxam–Gilbert sequencing at a time when automated sequencing machines were not yet available. Kreitman (1983) uncovered 43 SNPs, only 1 of which was responsible for the two allozyme variants—fast (*Adh-f*) and slow (*Adh-s*)—previously found in nearly all natural populations, while the other 42 were silent polymorphisms in either coding or noncoding regions that had been previously invisible to protein electrophoresis. Apart from these SNP variants, four indel polymorphisms and two homopolynucleotide runs were found outside the coding region of the gene. These data uncovered an unforeseen wide spectrum of different types of genetic variants segregating in populations, and supported the view that most amino acid changes were selectively deleterious. Years after Kreitman’s revolutionary study, the advent of automated Sanger sequencing brought new variation data for dozens of genes in several species, including *Drosophila* (Powell 1997). These studies showed that levels of variation at silent sites vary among different taxa by less than a factor of 10 (compared to allozymes, which vary by <10^4^; see previous section), that SNPs outnumber all kinds of structural variants, and that transposable element (TE) insertions segregate as low-frequency polymorphisms. More recently, Leffler *et al.* (2012) have estimated genetic diversity levels by compiling polymorphism data across 167 species in 14 phyla, determining that autosomal nucleotide diversity varies by only two to three orders of magnitude, compared to the population census (*N*_c_, the actual number of individuals in a population), which probably varies by a factor of 10^8^–10^10^. Among the different ecological factors and life-history traits, reproductive strategy has been found to be strongly correlated with the genetic diversity of species (Leffler *et al.* 2012; Romiguier *et al.* 2014).

The data from resequencing studies are homologous and independent sequences (or haplotypes) sampled in a DNA region of interest (Kreitman 1983). In *D. melanogaster*, haplotypes can be obtained directly because we can extract single chromosomes using balancers, while they need to be inferred in other outbreeding organisms. The availability of these haplotypic sequences allowed the development of more powerful statistical metrics to quantify variation than did the previous generation of allozyme data (Table 1). On the one hand, one can estimate nucleotide diversity in the region by taking each nucleotide site as an independent unit (one-dimensional measures of variation). However, tests that only use information on the frequency distribution of segregating sites are clearly ignoring a significant source of information: associations between segregating sites, or the haplotype structure of the sample. It has been shown that nearby nucleotide sites are not independent of each other; instead, alleles are clustered in blocks from 100–150 bp (Huang *et al.* 2014; Grenier *et al.* 2015) to 2 kb in the *Drosophila* genome (Miyashita and Langley 1988; Langley *et al.* 2012), and >100 kb in the human genome (1000 Genomes Project Consortium 2015). This haplotype structure is influenced by recombination as well as selective and demographic forces, and it can be described by the use of multi-dimensional measures of genetic variation, such as estimators of linkage disequilibrium (LD) (Table 1). These multi-dimensional diversity measures provide key information on the history and evolution of a DNA region, including the effective recombination rate ρ *=* 4*N*_e_*r* underlying the region (where *N*_e_ is the effective population size and *r* is the recombination rate per locus) (Table 1) (Hudson 1987; Nordborg and Tavare 2002; McVean *et al.* 2004). Both one-dimensional and multi-dimensional diversity components are necessary for a complete description of sequence variation, and thus haplotypic data provide the maximum level of genetic resolution to make inferences about evolutionary history and about the evolutionary process. With all this rich data in hand and an extensive arsenal of population genetics statistics already available (Table 1), different software applications were developed to automate the data analyses, including DnaSP (Rozas and Rozas 1995) and PAML (Yang 1997), which are still widely used software packages for population genetics (Table 2).

**Table 1:** The arsenal of parameters for population genetics/genomics analyses: measures of nucleotide diversity, LD, and tests of selection

Measure/test	Description	References
Nucleotide diversity measures (uni-dimensional measures)		
*S*, *s*	Number of segregating sites (per DNA sequence or per site, respectively)	Nei (1987)
H, *η*	Minimum number of mutations (per DNA sequence or per site, respectively)	Tajima (1996)
*k*	Average number of nucleotide differences (per DNA sequence) between any two sequences	Tajima (1983)
π	Nucleotide diversity: average number of nucleotide differences per site between any two sequences	Jukes and Cantor (1969); Nei and Gojobori (1986); Nei (1987)
θ, θ*_W_*	Nucleotide polymorphism: proportion of nucleotide sites that are expected to be polymorphic in any suitable sample	Watterson (1975); Tajima (1993, 1996)
SFS	Site/allele frequency spectrum: distribution of allele frequencies at a given set of loci in a population or sample	Ronen *et al.* (2013)
LD (multi-dimensional association among variable sites) and recombination		
*D*	Coefficient of LD whose range depends of the allele frequencies	Lewontin and Kojima (1960)
*D*′	Normalized *D*, independent of allele frequencies	Lewontin (1964)
*R*, *R^2^*	Statistical correlation between pairs of sites	Hill and Robertson (1968)
*Z_nS_*	Average of *R*^2^ over all pairwise comparisons	Kelly (1997)
*Z_A_/ZZ*	*Z_A_* is the average of *R*^2^ only between adjacent polymorphic sites. *ZZ* is *Z_A_* minus *Z_nS_*, which is an estimate of the recombination parameter *r*	Rozas *et al.* (2001)
Four-gamete test	Measure of historical recombination under the infinite-sites model	Hudson and Kaplan (1985)
ρ	Population-scaled recombination rate ρ *=* 4*N*_e_*r* [computed, *e.g.*, by LDhat (Auton and McVean 2007) and LDhelmet (Chan *et al.* 2012)]	Hudson (1987)
Selection tests based on the allele frequency spectrum and/or levels of variability		
Tajima’s *D*	Number of nucleotide polymorphisms with the mean pairwise difference between sequences	Tajima (1989)
Fu and Li’s *D*, D*	Number of derived nucleotide variants observed only once in a sample with the total number of derived nucleotide variants	Fu and Li (1993)
Fu and Li’s *F*, F*	Number of derived nucleotide variants observed only once in a sample with the mean pairwise difference between sequences	Fu and Li (1993)
Fay and Wu’s *H*	Number of derived nucleotide variants at low and high frequencies with the number of variants at intermediate frequencies	Fay and Wu (2000)
Zeng’s *E*, θ*_L_*, *DH*	Difference between θ*_L_* and θ*_W_*: the first is sensitive to changes in high-frequency variants. DH is a joint test including Tajima’s *D* and Fay and Wu’s *H*	Zeng *et al.* (2006)
Achaz’s *Y*	Unified framework for θ estimators on the basis of the allele frequency spectrum	Achaz (2009)
Fu’s *F_S_*	Test based on the allele frequency spectrum	Fu (1997)
Ramos-Onsins’ and Rozas’ *R_2_*, *R_3_*, *R_4_*, *R_2E_*, *R_3E_*, *R_4E_*	Tests based on the difference between the number of singleton mutations and the average number of nucleotide differences	Ramos-Onsins and Rozas (2002)
CL, CLR	Genome scan for candidate regions of selective sweeps based on aberrant allele frequency spectrum	Nielsen *et al.* (2005)
Selection tests based on comparisons of polymorphism and/or divergence between different classes of mutation		
*d_N_/d_S_*, *K_a_/K_s_*	Ratio of nonsynonymous to synonymous nucleotide divergence/polymorphism (ω)	Li *et al.* (1985); Nei and Gojobori (1986)
HKA	Degree of polymorphism within and between species at two or more loci	Hudson *et al.* (1987)
MK	Ratios of synonymous and nonsynonymous nucleotide divergence and polymorphism	McDonald and Kreitman (1991)
Estimators derived from extensions of the MK test or the DFE		
NI	Neutrality index that summarizes the four values in an MK test table as a ratio of ratios	Rand and Kann (1996)
DoS	Direction of selection: difference between the proportion of nonsynonymous divergence and nonsynonymous polymorphism	Stoletzki and Eyre-Walker (2011)
α	Proportion of substitutions that are adaptive	Charlesworth (1994); Smith and Eyre-Walker (2002)
DFE-α	Fraction of adaptive nonsynonymous substitutions, robust to low recombination	Eyre-Walker and Keightley (2009)
ω*_A_*	Rate of adaptive evolution relative to the mutation rate	Castellano *et al.* (2016); James *et al.* (2016)
*K_a+_*	Rate of adaptive amino acid substitution (*K_a+_* = α*K_a_*)	Castellano *et al.* (2016)
$\hat{d},$ $\hat{b},$ $\hat{f},$ $\hat{\gamma },$ $\hat{\alpha }$	Fractions of five different selection regimes derived from an extension of the MK test: $\hat{d},$ fraction of new mutations that are strongly deleterious and do not segregate in the population; $\hat{b},$ fraction of new mutations that are slightly deleterious and segregate at minor allele frequency (MAF) <5%; $\hat{f},$ fraction of new mutations that are neutral, calculated after removing the excess of sites at MAF <5% due to slightly deleterious mutations; $\hat{\gamma },$ subset of $\hat{f}$ corresponding to recently neutral sites; $\hat{\alpha },$ fraction of new mutations that are adaptive, calculated after removing slightly deleterious mutations	Mackay *et al.* (2012)
*L*_HRi_	Proportion of adaptive substitutions lost due to HRi	Castellano *et al.* (2016)
*r*_opt_	Optimal baseline recombination, above which the genome is free of the HRi and thus *L*_HRi_ = 0	Mackay *et al.* (2012); Castellano *et al.* (2016)
Selection tests based on LD		
Hudson’s haplotype test	Detection of derived and ancestral alleles on unusually long haplotypes	Hudson *et al.* (1994)
B/Q	Based on LD between adjacent pairs of segregating sites, under the coalescent model with recombination	Wall (1999)
*iHS*	Integrated haplotype score, based on the frequency of alleles in regions of high LD	Voight *et al.* (2006)
LRH	Long-range haplotype test, based on the frequency of alleles in regions of long-range LD	Sabeti *et al.* (2002)
HS	Haplosimilarity score: long-range haplotype similarity	Hanchard *et al.* (2006)
EHH	Extended haplotype homozygosity: measurement of the decay of LD between loci with distance	Sabeti *et al.* (2002)
LDD	LD decay: expected decay of adjacent SNP LD at recently selected alleles	Wang *et al.* (2006)
SGS	Shared genomic segment analysis: detection of shared regions across individuals within populations	Cai *et al.* (2011)
GIBDLD	Detection of genomic loci with excess of identity-by-descent sharing in unrelated individuals as signature of recent selection	Han and Abney (2013)
XP-EHH	Long-range haplotype method to detect recent selective sweeps	Sabeti *et al.* (2007)
H12, H2/H1	Haplotype homozygosity	Garud *et al.* (2015)
Population differentiation and associated selection tests		
*G_ST_*	Analysis of gene diversity (heterozygosity) within and between subpopulations	Nei (1973)
*F_ST_*	Average levels of gene flow based on allele frequencies, under the infinite-sites model	Hudson *et al.* (1992b)
Bayesian *F_ST_*	Probability that a locus is subject to selection based on locus-specific population differentiation, using a Bayesian method	Foll and Gaggiotti (2008)
*G_ST_*, *H_ST_*, *K_ST_*	Different test statistics based on haplotype frequencies and/or the number of nucleotide differences between sequences	Hudson *et al.* (1992a)
*S_nn_*	Genetic differentiation of subpopulations based on haplotypic data	Hudson (2000)
*Phi_ST_*	Correlation of haplotypic diversity at different levels of hierarchical subdivision	Excoffier *et al.* (1992)
Strobeck’s *S*	Measure of population structure based on the comparison of the observed number of alleles in a sample to that expected when θ is estimated from the average number of nucleotide differences	Strobeck (1987)
XP-CLR	Cross-population composite likelihood ratio test, based on allele frequency differentiation across populations	Chen *et al.* (2010)
TLK, TF-LK	Original Lewontin–Krakauer test (TLK) and an extension (TF-LK), aimed at detecting selection based on the variance of *F_ST_* across loci	Lewontin and Krakauer (1973); Bonhomme *et al.* (2010)
LSBL	Locus-specific branch length, based on pairwise *F_ST_* distances	Shriver *et al.* (2004)
hapFLK	Detecting of selection based on differences in haplotype frequencies among populations with a hierarchical structure	Fariello *et al.* (2013)

**Table 2:** Selection of software available for population genetics/genomics analyses

	Released	Last version	Language	OS	Supported alignment formats	Supported SNP data formats
DnaSP	1995	5.10.1 (2010/03)	Visual Basic	MS Windows	FASTA, MEGA, NBRF/PIR, NEXUS, PHYLIP	HapMap
PAML	1997	4.8a (2014/08)	ANSI C	UNIX/Linux, MAC OSX, MS Windows	PHYLIP, NEXUS (limited support)	—
LAMARC	2001	2.1.10 (2016/01)	C++	UNIX/Linux, MAC OSX, MS Windows	PHYLIP, (own)	(own)
Arlequin	2005	3.5.2.2 (2015/08)	C++, R	UNIX/Linux, MAC OSX, MS Windows	(own)	(own)
VariScan	2005	2.0.3 (2012/07)	C++	UNIX/Linux, MAC OSX, MS Windows	MAF, MGA, XMFA, PHYLIP	HapMap
PLINK	2007	1.9 beta 3.38 (2016/06), 1.07 stable (2009/10)	C/C++	UNIX/Linux, MAC OSX, MS Windows	—	PED/MAP (own)
adegenet and pegas	2008; 2010	Adegenet, 2.0.1 (2016/02); Pegas, 0.9 (2016/04)	R	UNIX/Linux, MAC OSX, MS Windows	FASTA, NEXUS, PHYLIP, (own)	VCF, FSTAT, GENETIX, GENEPOP, STRUCTURE, (own)
PopGenome	2014	2.1.6 (2015/05)	R	UNIX/Linux, MAC OSX, MS Windows	FASTA, NEXUS, MEGA, MAF, PHYLIP, RData, (own)	VCF, SNP, HapMap, MS, MSMS
ANGSD	2014	0.911 (2016/03)	C/C++	UNIX/Linux	BAM, CRAM, MPILEUP	VCF, GLF, BEAGLE

DnaSP, http://www.ub.edu/dnasp/ (Rozas and Rozas 1995, 1997, 1999; Rozas *et al.* 2003; Librado and Rozas 2009; Rozas 2009); PAML, http://abacus.gene.ucl.ac.uk/software/paml.html (Yang 1997, 2007); LAMARC, http://evolution.genetics.washington.edu/lamarc/index.html (Kuhner 2006; Kuhner and Smith 2007); Arlequin, http://cmpg.unibe.ch/software/arlequin35 (Excoffier *et al.* 2005; Excoffier and Lischer 2010); VariScan, http://www.ub.edu/softevol/variscan (Vilella *et al.* 2005; Hutter *et al.* 2006); PLINK, http://pngu.mgh.harvard.edu/∼purcell/plink/ (Purcell *et al.* 2007); adegenet, http://adegenet.r-forge.r-project.org/ (Jombart 2008; Jombart and Ahmed 2011); pegas, http://ape-package.ird.fr/pegas.html (Paradis 2010); PopGenome, http://popgenome.weebly.com/ (Pfeifer *et al.* 2014); and ANGSD, http://www.popgen.dk/angsd (Korneliussen *et al.* 2014).

After >30 years of surveys of nucleotide variation in either particular loci (Kreitman 1983; Hasson *et al.* 1998; Balakirev and Ayala 2003a,b, 2004) or in 100s of genomic regions at a time (Andolfatto 2007; Hutter *et al.* 2007), very large numbers of sequences in many genes and species accumulated in the databases (Clark *et al.* 2016), and tools were developed to make use of these publicly available data to characterize genetic diversity at a large scale (Casillas and Barbadilla 2004, 2006; Casillas *et al.* 2005). However, even the largest compilations of surveys of genetic diversity were limited by the fact that they showed genetic diversity in particular sampled regions of the genome rather than providing unbiased genome-wide measurements. It was clear that the next natural step toward the characterization of genetic variation would be the resequencing of complete genomes.

### The current population genomics era

#### Genome variation:

Even though the term population genomics started to appear in the literature from the late 1990s in the context of large-scale polymorphism studies at multiple genomic loci (Black *et al.* 2001; Luikart *et al.* 2003), the pure sense of the term refers to the resequencing and analysis of complete genomes within and/or between populations. While this was economically prohibitive by Sanger sequencing in most cases, Drosophilists again pioneered the field by publishing one of the first large-scale population genomics studies in *D. simulans* (Begun *et al.* 2007) (note that in this case the lines had diverse origin, which implies that this was not a “pure” population genomics study in the sense that the individuals studied did not come from a single population).

During the last decade, the development of next generation sequencing (NGS) technologies (Metzker 2010; Goodwin *et al.* 2016) has allowed the deciphering of complete genome sequences of 100s of individuals in many populations of *Drosophila* (Langley *et al.* 2012; Mackay *et al.* 2012; Huang *et al.* 2014; Lack *et al.* 2015), as well as 10s to 1000s of individuals of other species (Durbin *et al.* 2010; Cao *et al.* 2011; Gan *et al.* 2011; 1000 Genomes Project Consortium 2012, 2015; Fawcett *et al.* 2014; Harpur *et al.* 2014; 1001 Genomes Consortium 2016). Data coming from these massive parallel sequencing methods differ from all previous variation data obtained by allozymes and Sanger sequences, both in the amount and accuracy of the data. We now need to deal with millions or billions of short sequencing reads that contain a relatively high proportion of erroneous nucleotides, and bioinformatics has become essential in addressing the specific needs of all the steps from data acquisition, quality checking, and analysis, as well as storage and representation. Specifically, even though the statistics to measure genetic variation have remained basically the same (Table 1), the availability of such massive data collections has obliged the development of new data formats and methods to be able to preprocess the data (*i.e.*, assemble or map the sequences against a reference and call nucleotide polymorphisms), to manage and represent huge amounts of nucleotide variation data, as well as to deal with new problems of fragmented, noisy data, including missing nucleotides (*i.e.*, regions of the genome not sequenced in one or more individuals, which implies that the sample size varies across the genome) or sequencing errors (*i.e.*, incorrectly typed nucleotides) (Chaisson *et al.* 2015).The variant call format (VCF) has emerged as the *de facto* standard to represent whole-genome variation data (Danecek *et al.* 2011), although whole-genome alignment formats are also used as input to population genomics analyses, including compressed binary alignment map (BAM) files. Table 2 compiles a selection of the population genetics/genomics software developed from the release of DnaSP two decades ago (Rozas and Rozas 1995), with newly developed software offering solutions to deal with the complexities and data types of the current genomics era.

The whole-genome sequencing of pools of individuals (Pool-seq) has recently emerged as an approach that provides population genomics data at considerably lower costs than the resequencing of separate individuals (Schlötterer *et al.* 2014). With the availability of custom-tailored software tools, Pool-seq gives reasonably reliable SNP calls while dropping both sequencing and library preparation costs. Some limitations of Pool-seq include the unequal representation of individuals in small pools, the more difficult detection of sequencing or alignment errors, or the inability to provide haplotype or LD information above the read length (Schlötterer *et al.* 2014). Pool-seq has been applied to *Drosophila* to study the genome-wide patterns of polymorphism and its relationship with recombination (Nolte *et al.* 2013), to characterize the genomic distribution and population frequencies of TEs (Kofler *et al.* 2012), and to detect selective sweeps (Nolte *et al.* 2013), among others. Other approaches based on NGS that have been designed to reduce the costs of resequencing populations include exome sequencing (Warr *et al.* 2015) and restriction site-associated DNA sequencing (Davey and Blaxter 2010; Andrews *et al.* 2016), although both strategies give biased representations of polymorphisms in the genome (polymorphisms in transcribed regions or in restriction sites, respectively).

All in all, while the main aim of population genomics is still the description and interpretation of genetic variation within and between populations (Lewontin 2002), the technological approaches of genetic diversity studies have revolutionized the field.

#### Genome recombination:

In parallel with the growing amount of population genomics data, increasingly more detailed estimates of the pattern of recombination rate along the genome are being provided. Fine-scale recombination estimates are essential not only to understand the molecular mechanism underlying variation in recombination but also to gain precise knowledge about the relationships between recombination and population genetics parameters to infer its relevance on genome evolution. The ability to detect linked selection, for example, depends crucially on the variance of the recombination rate across a genome.

In *D. melanogaster*, two new high-resolution recombination estimates have recently been added to the classical coarse recombination map based on genetic crosses (Fiston-Lavier *et al.* 2010). The first is a statistical approach that infers the historical population recombination parameter, ρ *=* 4*N*_e_*r*, from LD patterns at multiple sites across the genome (Hudson 1987). Numerous sophisticated and computationally intensive methods have been developed for estimating ρ (Lin *et al.* 2013). The software LDhat (McVean *et al.* 2002, 2004; Auton and McVean 2007) scales well to large data sets and it has been applied to estimate recombination rates in humans (McVean *et al.* 2004; Myers *et al.* 2005; Frazer *et al.* 2007; Durbin *et al.* 2010), *Drosophila* (Langley *et al.* 2012), and other species (Johnson and Slatkin 2009; Tsai *et al.* 2010; Auton *et al.* 2012; Axelsson *et al.* 2012). LDhat was developed in the context of patterns of genome variation and recombination in humans. However, the *Drosophila* genome contains a much higher density of SNPs and registers higher recombination rates. The model underlying LDhat assumes a neutrally evolving population of constant size. Contrary to humans, where the footprints of positive selection are rather sparse (Hernandez *et al.* 2011), *Drosophila* genomes undergo rampant adaptation (see section *Determinants of Patterns of Genome Variation*), which could invalidate the inferences of recombination of ρ based on LDhat (Reed and Tishkoff 2006; Stephan *et al.* 2006). For this reason, Chan *et al.* (2012) proposed a new computational method, LDhelmet, for estimating fine-scale recombination rates in *Drosophila*, which has shown to be robust to the effects of natural selection. LDhelmet has been applied to Langley *et al.*’s (2012) genome variation data of *D. melanogaster* to obtain a fine-scale recombination map of the genome (Chan *et al.* 2012).

Using an ingenious technique which integrates the power of classical genetics with NGS, Comeron *et al.* (2012) achieved the first integrated high-resolution description of the recombination patterns of both intragenomic and population variation. Recombinant advanced intercross lines (RAIL) were generated from 8 crosses among 12 wild-derived lines. RAIL females were individually crossed to *D. simulans* and the *D. melanogaster* haploid genome of single hybrid progeny was inferred using bioinformatics. A total of >100,000 recombination events at a resolution down to 2 kb were reported, distinguishing between crossing over (CO) and gene conversion (GC) events. CO rates exhibit highly punctuated variation along the chromosomes, with *hot* and *cold spots*, while GC rates are more uniformly distributed. This resource has become an essential data set for further population genetics studies dealing with recombination in this species (Campos *et al.* 2014; Comeron 2014; Huang *et al.* 2014; Kao *et al.* 2015; Castellano *et al.* 2016).

All three kinds of maps show patterns of recombination at different scales, showing substantial variation in different regions of the genome depending on the scale. Broad-scale maps of recombination give an overview of the distribution of recombination along each arm (Myers *et al.* 2005); while at the fine-scale recombination rate, variation has been shown to be widespread throughout the human and *D. melanogaster* genomes, across all chromosomes, and among populations. Recombination events cluster in narrow hot spots of around 2 kb (McVean *et al.* 2004; Myers *et al.* 2005; Frazer *et al.* 2007; Comeron *et al.* 2012). Fine-scale analyses relating selection and linkage implicitly assume that the recombination map is a fixed genome property. Consequently, linked selection could be obscured if polymorphism from one species is analyzed with recombination rates calculated from a different species (Cutter and Payseur 2013).

Recombination estimates of Fiston-Lavier *et al.* (2010) and Comeron *et al.* (2012) are integrated into the *D. melanogaster* recombination rate calculator (http://petrov.stanford.edu/cgi-bin/recombination-rates_updateR5.pl).

## The Theory: Population Dynamics of Genetic Variation

### The (nearly) neutral theory as the paradigm

At the time when the genetic diversity of populations was beginning to be assessed by electrophoretic methods, Motoo Kimura realized that the large amount of genetic variation uncovered in nature, together with the previous observation that genetic differences accumulate linearly with time (Zuckerkandl and Pauling 1965), would either impose too great a segregating load to be explained by balancing selection, as initially proposed by the balance hypothesis (Dobzhansky 1970; Ford 1971); or an unsurmountable substitutional load if directional positive selection was driving the amino acid substitutions observed in proteins. Kimura suggested a radical alternative explanation to account for the patterns of protein variation and substitution: the bulk of existing polymorphisms and fixed differences between species are selectively neutral (Figure 2A) and functionally equivalent. Under this model, the frequency dynamics of neutral variants in the population is determined by the rate of mutation and random genetic drift. This proposition was called the neutral theory of molecular evolution (Kimura 1968), and its principal assertions are enumerated in Box 1.

Genetic drift is the random sampling of gametes at each generation in a finite population, which results in a random fluctuation of allele frequencies across generations and the loss of genetic variation (Kimura 1968). In an idealized panmictic population with an equal contribution of individuals to reproduction (the so-called Wright–Fisher model), the strength of genetic drift is inversely proportional to *N*_c_. However, real populations typically depart from the Wright–Fisher assumptions in several respects; hence the concept of effective population size (*N*_e_), the size of the idealized Wright–Fisher population that would show the same amount of genetic diversity or other parameters of interest as the actual population.

By formulating a revolutionary new concept, Kimura’s neutral theory encapsulates molecular evolution in one of the most elegant mathematical expressions of science: $K=\mu_{0}$ (Box 1). This simple equation combines the three levels of variation from its origin to its substitution in the population [mutation (individual level), polymorphism (population level), and divergence (species level)] in the same unifying framework. If variants are neutral, the population level is irrelevant to molecular evolution, because the evolutionary rate depends on the mutational rate only; intrapopulation polymorphism is just a random walk of variants in their process to fixation or loss. The linear accumulation of substitutions over time predicted by the neutral theory is the basis of the molecular clock hypothesis, which considers that the number of substitutions among divergent sequences is a linear function of their divergence times.

A serious challenge posed to Kimura’s neutral theory was that rates of protein evolution are proportional to absolute time, in years, and not to generation time. Noting that population size is generally inversely proportional to generation time, Tomoko Ohta refined Kimura’s neutral theory by introducing a new class of mutation: *nearly neutral* mutations (Ohta 1973). Their fitness lies in the interval between Kimura’s strictly neutral mutations and strongly deleterious mutations, and they might account for an important fraction of all mutations (Figure 2B). Ohta’s (1973) nearly neutral theory predicts that nearly neutral mutations are mostly eliminated by natural selection in large populations, but that a substantial fraction of them behave as effectively neutral and are randomly fixed in small populations. As a result of this process, the strength of purifying selection acting on slightly deleterious mutations and the generation time effect compensate, and protein evolution is fairly insensitive to generation time, contrary to what happens in Kimura’s strictly neutral DNA. In the early 1990s, Ohta developed a model that included both slightly deleterious and slightly beneficial mutations (Ohta 1972; Ohta and Gillespie 1996) (Figure 2B), which predicted the following dynamics in the population (Li 1978):

Mutations with fitness effects much smaller in magnitude than 1/*N*_e_ (measured in the heterozygous state with the wild type, in the case of a diploid, randomly mating population) are considered effectively neutral (Figure 3A, gray), and their fate is basically at the mercy of genetic drift.

Note that in a small population, the range between −1/*N*_e_ and 1/*N*_e_ is larger than in a large population, and therefore there are more effectively neutral mutations. In contrast, in a large population most mutations are subject to some sort of natural selection. Therefore, the tight relationship between *s* and *N*_e_ nicely explains why the same mutation can behave as effectively neutral in one species with a small *N*_e_ [if *s* is within the range (−1/*N*_e_, 1/*N*_e_)], while it can be subject to selection in another species with a large *N*_e_ [because *s* is outside the range (−1/*N*_e_, 1/*N*_e_)]. In particular, as *N*_e_ increases, genetic drift becomes less important in determining the fate of new mutations, while natural selection becomes more powerful in the elimination of deleterious mutations and increasing the frequency of those that are advantageous, even if these have small *s*. *N*_e_ is thus the key parameter determining the relative importance of selection *vs.* genetic drift. The range |*N*_e_*s*| = 1 delimitates the decisive borderline: if *N*_e_*s* is <1, genetic drift dominates; if it is >1, selection dictates the fate of mutations.

Because of its simplicity, intelligibility, robustness, and feasible theoretical predictions about the expected pattern of molecular polymorphism and evolutionary rate; the (nearly) neutral theory of molecular evolution became tremendously attractive, enthroned as the universal stochastic null model against which to test any selective or alternative nonneutral hypothesis (Box 2 and Table 1).

Box 2. **Genome-Wide Signatures of Selection and Tests of Selection Based on Polymorphism and/or Divergence Data**

Looking for evidence of positive selection is a widely used strategy for identifying adaptive variants (Bamshad and Wooding 2003; Nielsen 2005; Vitti *et al.* 2013; Haasl and Payseur 2016) and quantifying the impact of selection on the genome. During the process of fixation of adaptive variants, linked neutral variation is dragged along with the selected site; thus reducing the levels of genetic diversity in the region, while simultaneously new mutations accumulate in the region (see section *Genetic draft as a selectionist alternative to the neutral theory*). These mutations represent most of the genetic variation in the region depauperated by the selective sweep, and their initial frequency is low, so that a region harboring a positively selected variant will also harbor an excess of rare derived alleles. Furthermore, if an allele influenced by recent positive selection increases in frequency faster than local recombination reduces the range of LD between the allele and linked markers, then the region will also show unusually long-range LD (Nielsen 2005; Franssen *et al.* 2015; Garud *et al.* 2015; Garud and Petrov 2016). As a whole, natural selection leaves signatures in the genome that can be used to identify the regions that have been selected, including:

1. A reduction in the genetic diversity.
2. A skew toward rare derived alleles.
3. An increase in the LD.

**Figure:**
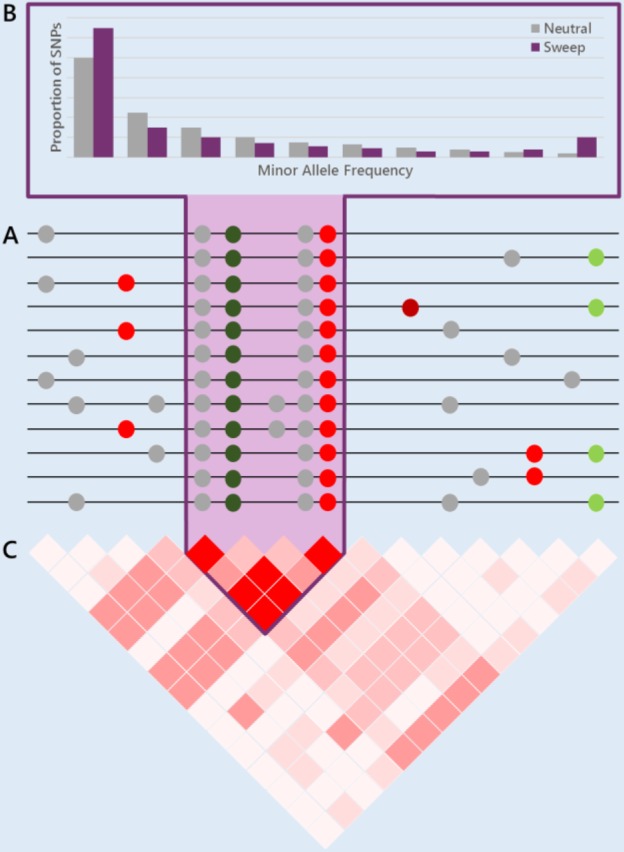
**Signatures of a selective sweep in the genome** (A) A reduction in genetic diversity, (B) a skew toward rare derived alleles, and (C) an increase in LD (see text for details). Colored ● reflects different classes of mutations according to their fitness effects: maroon, strongly deleterious (very infrequent, in their way to elimination by natural selection); red, slightly deleterious; gray, neutral; light green, slightly advantageous; dark green, advantageous. Note that in the region of the selective sweep (purple), an advantageous mutation has been driven to fixation together with its linked neutral and nearly neutral variants. In this region, genetic diversity is reduced, most polymorphisms are shared among different chromosomes (high LD), while recently arisen mutations are still at low frequency (gray ● present in two chromosomes).

Since the signatures of selection depend greatly on the local rate of recombination, variable recombination along the genome renders the detection of selection difficult (Hudson and Kaplan 1995). The confounding effects of both recombination and demography in the patterns of genetic variation challenge the identification of regions in the genome showing true signatures of adaptive evolution (Teshima *et al.* 2006; Bachtrog and Andolfatto 2006). Furthermore, all of these signatures quickly dissipate with time (Kim and Stephan 2002; Nielsen *et al.* 2005); therefore, this approach can only identify very strong and recent adaptive events. However, the wealth of nucleotide polymorphism data that has become available during the past few years has provided an increased opportunity to conduct genome scans for selection and many instances of selective sweeps have been found in *Drosophila* (Schlötterer 2002; Kauer *et al.* 2003; Akey *et al.* 2004; Wiehe *et al.* 2007; Pool *et al.* 2012; Brand *et al.* 2013; Garud *et al.* 2015), as well as other species (Haasl and Payseur 2016).

Another selective process also reduces the level of genetic variation in the region: *BGS* (*i.e.*, the recurrent elimination of chromosomes carrying strongly deleterious mutations) (Charlesworth *et al.* 1993; Braverman *et al.* 1995; Charlesworth *et al.* 1995). The effect in this case is to reduce the number of chromosomes that contribute to the next generation, thus reducing the levels of genetic diversity in the region. In contrast to a hitchhiking event, it neither skews the distribution of rare polymorphisms, nor generates LD blocks. In this sense, the result is identical to that of a reduction in population size, except that the reduction applies not to the genome as a whole, but to a tightly linked region (Charlesworth *et al.* 1993). Finally, balancing selection and local adaptation leave other particular signatures of selection in the genome that include haplotypes at an intermediate frequency, with strong population differentiation, and a high level of LD with respect to variants at surrounding sites (Charlesworth *et al.* 1997).

Several tests have been developed to quantify the amount of selection in the genome using polymorphism and/or divergence data (Table 1). We will focus here on standard tests that have been the basis of today’s most sophisticated statistical methods to spot genomic regions modeled by natural selection, and we direct the reader to Vitti *et al.* (2013) for a more comprehensive review of all the methods available.

***d*_*N*_*/d*_*S*_ (or *K*_*a*_*/K*_*s*_) ratio**

Assuming that silent substitutions are neutral, if advantageous mutations have been frequent among nonsynonymous sites and have spread through the population faster than neutral mutations, then the rate of nonsynonymous substitution—*d*_*N*_ or *K*_*a*_—will be significantly greater than the rate of silent substitution—*d*_*S*_ or *K*_*s*_. On the other hand, if replacement substitutions are mostly removed by negative selection, *d*_*N*_ will be significantly lower than *d*_*S*_. Thus, the ratio ω = *d*_*N*_*/d*_*S*_ (Yang and Bielawski 2000) is used as a common measure of functional constraint: *d*_*N*_*/d*_*S*_ = 1 under neutrality, is <1 under functional constraint, and is >1 under positive selection. Note that the method assumes that (1) synonymous substitutions are neutral; and (2) all substitutions have the same biological effect, which might not be the case. This test is conservative because most nonsynonymous mutations are expected to be deleterious and *d*_*N*_ tends to be much lower than *d*_*S*_. Thus, the proportion of adaptive substitutions needs to be high for adaptive evolution to be detectable using this method.

**The MK test**

The MK test (McDonald and Kreitman 1991) was developed as an extension of the Hudson–Kreitman–Aguadé test (Hudson *et al.* 1987). It was designed to be applied to protein coding sequences, combining both between-species divergence (*D*) and within-species polymorphism (*P*) sites, and categorizing mutations as synonymous (*P*_*s*_, *D*_*S*_) and nonsynonymous (*P*_*n*_, *D*_*N*_). If all mutations are either strongly deleterious or neutral, then *D*_*N*_*/D*_*S*_ is expected to roughly equal *P*_*n*_*/P*_*s*_. In contrast, if positive selection is operating in the region, adaptive mutations rapidly reach fixation and thus contribute relatively more to divergence than to polymorphism when compared to neutral mutations, and then *D*_*N*_*/D*_*S*_ > *P*_*n*_*/P*_*s*_. We can summarize the four values as a ratio of ratios termed the neutrality index (NI) as NI = [(*P*_*n*_*/P*_*s*_)*/*(*D*_*N*_*/D*_*S*_)] (Rand and Kann 1996) and quantify the significance of the effect using a simple 2 × 2 contingency table. The MK test can also be extended to other functional regions of the genome, such as noncoding DNA, assuming that one of the two classes compared evolves neutrally (Casillas *et al.* 2007; Egea *et al.* 2008).

Furthermore, assuming that adaptive mutations contribute little to polymorphism but substantially to divergence, data from an MK test can be easily used to estimate the proportion of nonsynonymous substitutions that have been fixed by positive selection as α *=* 1 − (*D*_*S*_*P*_*n*_*/D*_*N*_*P*_*s*_) (Charlesworth 1994; Smith and Eyre-Walker 2002). However, this estimate can be easily biased by the segregation of slightly deleterious nonsynonymous mutations (Eyre-Walker 2002) and different demographic histories. If the population size has been relatively stable, α is underestimated, because slightly deleterious mutations tend to contribute relatively more to polymorphism than they do to divergence when compared with neutral mutations. On the contrary, slightly deleterious mutations can lead to an overestimate of α if population size has expanded, because those slightly deleterious mutations that could become fixed in the past by genetic drift due to the small population size only contribute to divergence (Eyre-Walker 2002). Because these slightly deleterious mutations tend to segregate at lower frequencies than do neutral mutations, they can be partially controlled for by removing low-frequency polymorphisms from the analysis (Fay-Wycoff-Wu method, FWW) (Fay *et al.* 2001). However, the FWW method is still expected to lead to biased estimates, unless the DFE is strongly L-shaped or the level of adaptation is very high (Charlesworth and Eyre-Walker 2008). Eyre-Walker and Keightley (2009) developed the DFE-α as an unbiased estimate of the percentage of adaptation occurring in the genome, even in regions of little or no recombination. They estimated α by simultaneously estimating the DFE at selected sites from the SFS and the number of adaptive substitutions.

**The coalescence theory**

The first theoretical models in population genetics simulated the evolution of populations *forward-in-time*, trying to understand how a population subject to mutation and genetic drift, and maybe recombination, natural selection, and gene flow, will evolve from a past or present time toward the future (Crow and Kimura 1970). The coalescence theory (Kingman 1982a,b, 2000) follows a different approach in which a present sample from a population is traced back to a single ancestral copy, known as the most recent common ancestor. It is thus a *backward-in-time* stochastic model that relates genetic diversity in a sample to demographic history of the population from which it was taken. In this process, coalescent events are represented as a gene genealogy. Many software applications have been developed to simulate data sets under the coalescent process, as well as to infer population genetics parameters such as population size and migration rates from genetic data [see, *e.g.*, LAMARC (Kuhner 2006; Kuhner and Smith 2007) in Table 2].

### The distribution of fitness effects

Typically, we categorize a new mutation that enters the population as being neutral when it does not affect the fitness of the individual bearing it, deleterious when the mutation is detrimental (or even lethal), or advantageous when the mutation increases the fitness of the individual. However, there is a continuum of selective effects, the distribution of fitness effects (DFE) (Eyre-Walker and Keightley 2007; Lanfear *et al.* 2014), such that the effects of mutations range from those that are strongly deleterious (Figure 3A, maroon), weakly deleterious (Figure 3A, red), effectively neutral (Figure 3A, gray), and weakly (Figure 3A, light green) and highly advantageous (Figure 3A, dark green) mutations. In fact, there is not a unique DFE that applies to all nucleotide sites in the genome; each type of nucleotide, depending on the functional class to which it belongs, has its own DFE.

A number of mathematical distributions with two parameters, including the normal, lognormal, and gamma distributions, have been used to model the DFE; although a distribution with a good fit to the data has not yet been resolved (Loewe *et al.* 2006; Loewe and Charlesworth 2006; Eyre-Walker and Keightley 2007; Keightley and Eyre-Walker 2010; Tamuri *et al.* 2012; Kousathanas and Keightley 2013; Lanfear *et al.* 2014). One procedure to estimate the DFE is by comparing the levels of synonymous and nonsynonymous variability across species with very different *N*_e_’s. The extent to which the levels of nonsynonymous variability differ compared to the corresponding difference in the levels of synonymous variability (assumed to evolve neutrally), reflects the nature of the DFE on nonsynonymous variants (Loewe *et al.* 2006; Haddrill *et al.* 2010). The results of these and other studies in *Drosophila*, with *N*_e_ in the millions, suggest a wide and highly skewed DFE toward weakly and strongly deleterious variants with values of the strength of selection, *N*_e_*s*, ranging from 1–10 (Sawyer *et al.* 2003), ∼12 (Keightley *et al.* 2016), ∼40 (Andolfatto 2007), 350–3500 (Eyre-Walker 2006, reanalyzing Andolfatto’s 2005 data), ∼2000 (Li and Stephan 2006; Jensen *et al.* 2008), to ∼10,000 (Macpherson *et al.* 2007). These disparate estimations are in part due to several assumptions made by the different methods, such that advantageous mutations are weakly selected (Sawyer *et al.* 2003), or that the correlation between diversity and recombination rate is solely due to genetic hitchhiking (Eyre-Walker 2006, reanalyzing Andolfatto’s 2005 data). In other cases, the differences are due to subtler differences in the methodology used, such as the size of the genomic windows considered in the analyses (Andolfatto 2007; Macpherson *et al.* 2007), or the misassignment of the ancestral state in the unfolded site frequency spectrum (SFS) (Keightley *et al.* 2016). Interestingly, Sattath *et al.* (2011) reveal a substantial variation in the fitness effects of adaptive amino acid substitutions in *Drosophila*. According to their model, a minority of amino acid substitutions appears to have had large selective effects and account for most of the reduction in diversity, while the majority of amino acid substitutions are only weakly selected. This finding might also account for the disparate estimates of the strength of selection published for this species.

The rate of molecular evolution (*K*) is the speed at which genome changes are incorporated (fixed) in a given species in each generation. If genome divergence is the final evolutionary consequence of the molecular population dynamics, then *K* informs about the rhythm at which species diverge through their evolutionary time (Figure 3). *K* is the fixation rate averaged over all mutations entering the population. Specifically, mutations enter the population at a rate 2*Nμ* (the mutation rate *μ* is per site per generation, and in a diploid population there are 2*N* potential chromosomes to mutate) (Figure 3C). Each of these new mutations have a given selection coefficient *s* that is determined by its fitness effect on the individual (DFE, Figure 3A). All mutations with this *s*, *f*(*s*), have a probability of fixation that depends both on the population census and the effective population size, in addition to the selection coefficient, *u*(*N*, *N*_e_, *s*) (thus contributing to the divergence between species) (Kimura 1957; Figure 3B). *s* potentially ranges from −∞ to +∞ (sometimes scaled from −1 to 1), so the overall molecular evolutionary rate (*K*) taking into account all mutations is determined by the general expression:

$$K=2N\mu \int_{-\infty }^{\infty }u\left(N,N_{e},s\right)f\left(s\right)ds.$$

Now, let us consider the assumptions of the neutral theory that mutations are either effectively neutral (*s* ≈ 0, the fraction *μ*_0_) or strongly deleterious. For simplicity, let us also consider that the effective population size equals the population census (*N*_e_ = *N*). The general expression simplifies to $K=2N\left[{µ}_{0}u\left(N,s=0\right)+\left(µ-{µ}_{0}\right)u\left(N,s=-\infty \right)\right]$. If the probability of fixation of strongly deleterious mutations is null [u(N,s=−∞)=0], then K=2Nμ0u(N,s=0)=2Nµ0 12N=µ0, getting back the Kimura’s minimalist equation *K* = *μ*_0_. Note that the probability of fixation of a neutral mutation equals its initial frequency in the population, u(N,s=0)=12N.

### Genetic draft as a selectionist alternative to the neutral theory

Even though the Kimura’s neutral theory predicts a linear relationship between the extent of genetic diversity and population size (θ *=* 4*N*_e_μ; Box 1), data unambiguously show that the wide range in population sizes over all species is not linearly reflected in their relatively similar genetic diversities (see sections *The allozyme era: setting the stage for the neutralist–selectionist debate* and *The nucleotide sequence era*). Smith and Haigh (1974) proposed *genetic hitchhiking* as an explanation for the apparent population size paradox. In this process, neutral alleles that are sufficiently tightly linked to a favorable mutation go to fixation along with the favorable mutation, resulting in a reduction of linked genetic variation (what was later called a *selective sweep*; Berry *et al.* 1991).

In the late 1980s, when allozyme polymorphism studies were replaced by DNA-based markers, genetic variation was shown to be reduced in regions of low recombination in *Drosophila*, such as in the centromeres or within chromosome rearrangements (see section *The nucleotide sequence era*). After excluding mutation as the explanation for this correlation, Begun and Aquadro (1992) invoked recurrent natural selection to explain the observed pattern: within-species variation had to be more rapidly eliminated in regions of low recombination. John Gillespie revised the hitchhiking hypothesis and developed a stochastic model of the process he calls *genetic draft* (Gillespie 2000a,b, 2001). Like genetic drift, draft removes genetic variation from the population, although in this case the effect increases with population size. In particular, as *N*_e_ increases, genetic drift is less effective in removing alleles from the population and genetic variation tends to increase. But at the same time, more adaptive mutations occur (since there are more alleles to mutate) and selection is more prevalent, so more genetic hitchhiking events occur that reduce the level of genetic diversity in the region linked to the event. Once *N*_e_ is sufficiently large, genetic draft dominates and genetic variation becomes insensitive to population size. Thus, through this alternative model, Gillespie was able to uncouple population size and the levels of genetic diversity (Gillespie 2004; Lynch 2007).

The genetic draft effect is more prominent in regions of the genome with reduced recombination, where hitchhiking events leave a trace in a larger region which is linked to the selected variant. In the case of the mitochondrial chromosome (mtDNA), the levels of recombination are much lower than in the nuclear DNA, and this tightly linked region spans the whole chromosome. For this reason, selectively advantageous mutations that arise in the mtDNA constantly remove all previously existing variation in the chromosome and levels of mtDNA diversity appear to be similar across distant species, independently of their population size (Bazin *et al.* 2006). As a result, ∼58% of amino acid substitutions in invertebrate mtDNA are selectively advantageous (∼12% in vertebrate mtDNA), and mtDNA diversity is essentially unpredictable by population size and may only reflect the time since the last hitchhiking event rather than population history and demography (Bazin *et al.* 2006).

Thus, a byproduct of selection acting on an adaptive variant is the reduction of nearby genetic diversity. Charlesworth *et al.* (1993) proposed that a similar effect should be observed around deleterious variants, a process known as background selection (BGS) (Box 2). Selective sweeps are expected to dominate when selection is strong, and adaptive mutations are common. On the contrary, BGS will predominate when selection is relatively weak and mutations are recessive. While both mechanisms have long been proposed as being responsible for wiping out the expected relationship between genetic diversity and population size, *i.e.*, Lewontin’s paradox, it has not been until recently that a wealth of population genomics data from a wide range of species has been available to empirically test the effects of linked selection on the surrounding levels of genetic diversity. Corbett-Detig *et al.* (2015) have modeled the expected reduction in neutral diversity by BGS and hitchhiking under different recombination rates for 40 different eukaryotic species, showing that while the effects of selection on neutral diversity can be substantial, they vary between species according to *N*_c_; *i.e.*, natural selection has a greater impact on the levels of linked neutral variation in species with large *N*_c_ than in those with small *N*_c_. It is concluded that in species with a large population size, such as *D. melanogaster*, natural selection truncates the upper tail of the distribution of neutral variation. This study provides direct empirical evidence that natural selection in large populations constrains the levels of neutral genetic diversity and contributes to explain the long-standing paradox of population genetics.

In one of the most attractive hypotheses of the last decade, Michael Lynch (2006, 2007) proposes that not only genetic variation, but also the very complexity of the genome is a consequence of population genetic processes. In very large populations, selection is so efficient that genomes cannot leave their adaptive peak to investigate new landscapes. In contrast, in small eukaryotic populations, inefficient selection allows the genome to accumulate slightly deleterious mutations that will eventually be the source for adaptive innovations. Thus, the complexity of the eukaryotic genome would be initiated by nonadaptive processes, which in turn would provide a new substrate to secondarily build novel forms of organismal complexity through the action of natural selection.

## Patterns of Genome Variation

The immense outpouring of genome variation data precipitated by NGS techniques has made the empirical aim of population genetics a reality (Lewontin 1991). Detailed genome-wide descriptions of the nucleotide, indel, and TE variation patterns of several model species are already available [for *D. melanogaster* (Langley *et al.* 2012; Mackay *et al.* 2012; Huang *et al.* 2014; Lack *et al.* 2015), yeasts (Liti *et al.* 2009; Strope *et al.* 2015), *A. thaliana* (Cao *et al.* 2011), *C. elegans* (Andersen *et al.* 2012), as well as humans (Durbin *et al.* 2010; 1000 Genomes Project Consortium 2012, 2015)]. Population genetics studies prior to the population genomics era were based on fragmentary and often biased nonrandom samples of the genome, but the genomic dimension has provided us with the complete variational census along any chromosome and functional region of the genome. Population genomics surveys have allowed refining, improving, and clarifying patterns and processes of nucleotide variation previously studied in smaller data sets (Smith and Eyre-Walker 2002; Andolfatto 2005; Presgraves 2005; Casillas *et al.* 2007; Sackton *et al.* 2009; Sella *et al.* 2009); but more importantly, the genome perspective has provided qualitative new insights about the action of selection and the limits imposed by the architecture of the genome on adaptation. The 40-year-long neutralist–selectionist debate has shifted toward a new perspective: recombination has become a decisive parameter, determining the relative importance of genetic drift *vs.* genetic draft at the intragenomic variation level.

### The inquiry power of population genomics

The first step in any population genomics study is estimating the parameters that capture the evolutionary properties of the analyzed sequences (*e.g.*, polymorphism and divergence measures, proportion of adaptive fixations; see Table 1). This parameter inventory confers a large integrative capacity in both the level of genomic explanation and in the multi-omics level.

At the genomic level, these population parameters can be correlated throughout the genome with other genomic variables such as the local recombination rate, GC bias, gene density, chromosome arm, or chromosomal region, to assess the relative impact of the genomic determinants of genetic variation. Which part of the within-genome variation is ascribable to each genomic determinant? How much do these genomic variables constrain the adaptive capacity of the genome? Especially relevant is the interaction between selection and recombination and its relationship with the Hill–Robertson interference (HRi) process (see *Pervasive selection and the HRi*).

At the multi-omics level, the patterns of genomic diversity can be correlated with annotations of large sets of “-omics” data (*e.g.*, transcriptomics, epigenomics) allowing the integration of large sets of -omics data to gain a global (systemic) view of the causes and evolutionary and functional effects of genome variation (Wagner 2008; Loewe 2009).

### Population genomics in *Drosophila*

The first population genomics study in a *Drosophila* species was carried out by Begun *et al.* (2007) in *D. simulans*. Seven inbred lines of diverse origin were sequenced by whole-genome shotgun and the genome assemblies were compared with the sequences of the closely related species, *D. melanogaster* and *D. yakuba*. Despite the low number of lines, large-scale fluctuations of polymorphism and divergence were found along chromosome arms, there was significantly less polymorphism and faster divergence on the *X* chromosome, a correlation between recombination rates and sequence variation was found, and there was evidence of adaptive protein evolution at 19% of 6702 analyzed genes. The study provided the first direct genome-wide evidence showing that natural selection is pervasive in the genome of a *Drosophila* species.

In *D. melanogaster*, a preliminary study by Sackton *et al.* (2009) surveyed natural variation in nine strains from African (*n* = 3) and North American (*n* = 6) populations based on low-coverage sequencing. Later, two ambitious population genomics projects have allowed two independent population genomics studies in the same species. The DGRP (Mackay *et al.* 2012), a community resource for the analysis of population genomics and quantitative traits, has fully sequenced 158 inbred lines (Mackay *et al.* 2012), later extended to a total of 205 lines (Huang *et al.* 2014), derived from a North American natural population (RAL). From a pure population genetics perspective, the availability of 205 deep-coverage genomes from a single natural population represented an unprecedented opportunity to perform the most comprehensive population genetics study done so far in any species. Using an integrated genotyping strategy, 4,853,802 SNPs and 1,296,080 non-SNP variants were identified. The population genome browser, PopDrowser (Ràmia *et al.* 2012), has been designed for visualizing and querying the summary statistics, LD parameters, and several neutrality tests along the chromosome arms of the DGRP sequence data. The DPGP (Langley *et al.* 2012) independently analyzed the genome-wide polymorphism of two natural populations of *D. melanogaster*: 37 DGRP lines and 6 from a population of Malawi (Africa, MW data). The genome sequences of *D. simulans* and *D. yakuba* (Drosophila 12 Genomes Consortium *et al.* 2007) were used to estimate the divergence pattern. Variation patterns along the chromosome arms were measured (1) through different nonoverlapping window-sized units, and (2) for different DNA functional regions [coding (synonymous and nonsynonymous), 5′ and 3′ UTR, intron, and intergenic]. Here, we will focus on the following results of these studies: (1) Description of the patterns of polymorphism and divergence (nucleotide, indels, and TE) along chromosome arms and for different functional classes; (2) mapping natural selection throughout the genome; (3) local recombination rate and patterns of variation and selection; and (4) quantifying the cost of linked selection, *i.e.*, the Hill–Robertson effect.

#### Nucleotide variation:

Nucleotide heterozygosity π is around 41% larger in the ancestral geographical MW population (π = 0.00752) than in the North America RAL population (π = 0.00531). The genome patterns of polymorphism differ manifestly along chromosome arms, mainly between centromeric *vs.* noncentromeric regions within autosome arms; while divergence is rather homogeneous along the whole chromosome arms. Autosomal nucleotide diversity is reduced on average two- to fourfold in centromeric regions relative to noncentromeric regions, as well as at the telomeres; whereas it is relatively constant along the *X* chromosome. Average polymorphism on the *X* chromosome is reduced relative to the autosomes in the RAL population, but not in the MW population. Genes on the *X* chromosome evolve faster than autosomal genes (*X*:autosome ratio = 1.131 in the RAL population). Common inverted and standard karyotypes are genetically divergent and account for most of the variation in relatedness among the DGRP lines (Huang *et al.* 2014).

The pattern of polymorphism and divergence by site functional class is consistent within and among chromosomes (π_synonymous_ > π_intron_ > π_intergenic_ > π_UTR_ > π_nonsynonymous_), in agreement with previous studies on smaller data sets (Andolfatto 2005; Sella *et al.* 2009). Polymorphism levels between synonymous and nonsynonymous sites differ by an order of magnitude (π_synonymous_ = 0.0120; π_nonsynonymous_ = 0.0016) (Mackay *et al.* 2012; Barrón 2015). Polymorphism and divergence patterns within the site functional classes generally follow the same patterns observed overall.

#### Indel variation:

A measure analogous to nucleotide heterozygosity, π_indel_, is used to describe indel polymorphism (Huang *et al.* 2014). This measure does not take indel size into account. The pattern of π_indel_ along chromosomes is similar to that of SNP nucleotide diversity. There is a strong positive correlation between indel and nucleotide diversity for all chromosome arms (Massouras *et al.* 2012; Huang *et al.* 2014).

Evolutionarily derived deletions outnumber insertions, the deletion:insertion ratio for *D. melanogaster* is 2.2:1. This estimate is consistent with previous estimates that indicate a bias toward higher deletion than insertion rates (Petrov 2002; Ometto *et al.* 2005; Assis and Kondrashov 2012; Leushkin *et al.* 2013). There are on average 60% fewer deletions and 74% fewer insertions on the *X* chromosome than on the major autosomal chromosomal arms, consistent with stronger selection against indels on the *X* chromosome (see below).

#### TE variation:

Barrón *et al.* (2014) have recently reviewed different evolutionary models to explain the diversity of TEs present in the *Drosophila* genome, where they account for ∼20% of the genomic sequence. Most TEs are present at low population frequencies, especially those found in high-recombining regions of the genome (Bartolomé *et al.* 2002; Lee and Langley 2010; Petrov *et al.* 2011; Kofler *et al.* 2012; Cridland *et al.* 2013), and reside mainly outside exons or untranslated regions.

### Mapping natural selection throughout the genome

By applying the standard (McDonald and Kreitman 1991) and extended (Egea *et al.* 2008; Mackay *et al.* 2012) McDonald–Kreitman (MK) tests (Box 2 and Table 1), natural selection has been mapped along the genome both for overlapping sliding windows and in coding or noncoding functional regions for different selection regimes (Mackay *et al.* 2012). Results showed that natural selection is pervasive along the *D. melanogaster* genome, and that the relative importance of different selection regimes depends on both the site classes and the genome regions considered.

#### Prevalence of weakly negative selection:

For nucleotide variation, both the SFS of variants and the test comparing polymorphism and divergence (MK test and extensions) show large numbers of segregating sites undergoing weak negative selection. There is an excess of rare alleles with respect to the neutral expectation, both for SNPs and indels (Mackay *et al.* 2012; Huang *et al.* 2014). Selection regimes on a gene region differ according to the site class. Averaged over the entire genome, 58.5% of the segregating sites are neutral or nearly neutral, 1.9% are weakly deleterious, and 39.6% are strongly deleterious. Nonsynonymous sites are the most constrained (77.6% are strongly deleterious). However, these proportions vary between the *X* chromosome and the autosomes, site classes, and chromosome regions. The inferred pattern of selection differs between autosomal centromeric and noncentromeric regions: strongly deleterious mutations are reduced in the centromeric regions for all site categories; but no such effect is found in the *X* chromosome, which exhibits a higher proportion of strongly deleterious alleles for all site classes and regions.

**Figure 4:**
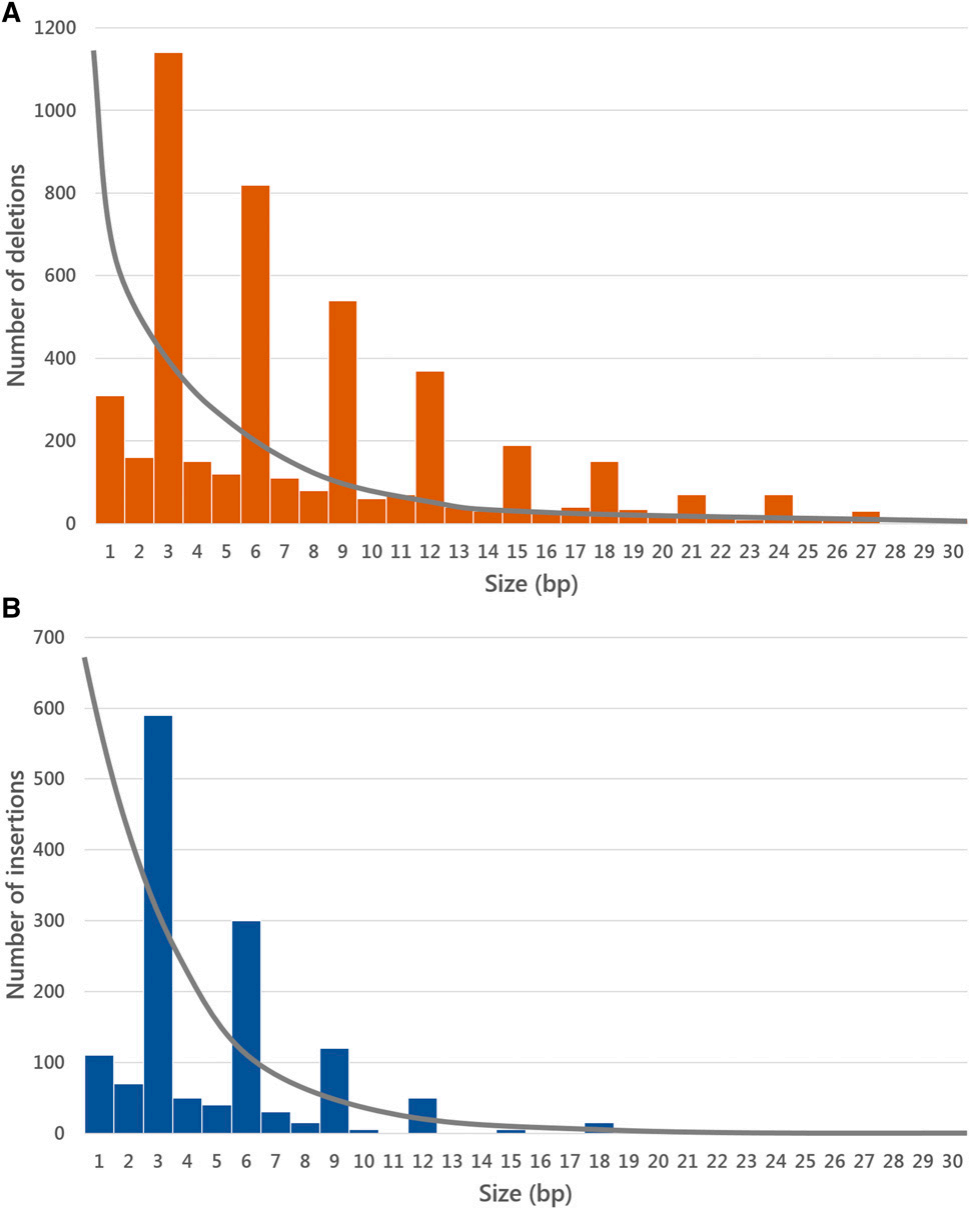
The footprint of deleterious selection on indel variation. Indel size distribution of (A) deletions and (B) insertions in coding regions (bars) and short introns (for comparison, gray line). The size distribution of indels in coding regions has discrete peaks for indel sizes in multiples of 3 bp. This remarkable pattern is a classroom example of the footprint that natural selection against frameshifting indels leaves, compared to a more relaxed selection for insertions and deletions spanning complete codons or short introns. Data from Massouras *et al.* (2012) and Huang *et al.* (2014).

The distributions of indel size are similar for 3′ and 5′ UTRs, large and small introns, and intergenic regions; while the size distribution of indels in coding regions has discrete “peaks” for indel sizes in multiples of 3 bp (Figure 4). This is a vivid classroom example of the footprint of natural selection due to strong negative selection against frameshifting indels compared to a more relaxed selection for insertions and deletions spanning complete codons, which has been reported both in the *Drosophila* DGRP lines (Massouras *et al.* 2012; Huang *et al.* 2014) and in humans (Montgomery *et al.* 2013).

Relative to presumed neutral variants (synonymous SNPs and SNPs in small introns), all deletion classes have an excess of low frequency-derived alleles on all chromosomes; this phenomenon is not observed for insertions. These results suggest that natural selection acts differently on insertions and deletions, with stronger purifying selection on deletions (Petrov 2002; Assis and Kondrashov 2012; Leushkin *et al.* 2013). This is consistent with the mutational equilibrium theory for genome size evolution (Petrov 2002), where optimal genome size is maintained by purifying selection on small deletions and less selection on long insertions, compensating for sequence loss.

Two main models of TE dynamics, namely the *transposition-selection balance* model and the *burst transposition* model have been proposed to account for the maintenance of TEs in populations. The former model postulates an equilibrium between an increase in copy number by a constant transposition rate and the elimination of TE copies from the population by purifying selection (Charlesworth 1983; Charlesworth *et al.* 1994). The observed low TE population frequencies support this model (González *et al.* 2008; Kofler *et al.* 2012; Cridland *et al.* 2013; Blumenstiel *et al.* 2014). The elimination is mainly carried out by removing copies that alter nearby genes or regulatory regions (Finnegan 1992; McDonald *et al.* 1997), or subsequent chromosomal rearrangements that lead to inviable gametes by ectopic recombination (Montgomery *et al.* 1987).

The burst transposition model assumes that some families can undergo periods of transposition bursts during which purifying selection might not be so intense (Kidwell 1983; Daniels *et al.* 1990), such that recently active families (*e.g.*, LTR families) show low population frequencies compared to long-time inactive families (*e.g.*, non-LTR families) with fixed copies (Bergman and Bensasson 2007; Blumenstiel *et al.* 2014). As a result, TE age determines TE frequency to a large extent, together with other explanatory variables such as recombination, TE length, or distance to the nearest genes (Blumenstiel *et al.* 2014).

#### Wide evidence of adaptive evolution:

Nucleotide variation shows substantial evidence for positive selection (adaptive fixation) in autosomal noncentromeric regions and in the *X* chromosome. Estimates of α, the proportion of adaptive substitution from the standard and generalized MK test, indicate that on average 25.2% of the fixed sites between *D. melanogaster* and *D. yakuba* are adaptive, ranging from 30% in introns to 7% in UTR sites. The majority of adaptive fixations on autosomes occur in noncentromeric regions. α is two to four times larger for the *X* chromosome than for autosomes. The pattern holds for all site classes, in particular nonsynonymous sites and UTRs, as well as individual genes, and it is not solely due to the autosomal centromeric effect. In indels, there is a slight excess of high frequency-derived insertions compared to SNPs in all chromosomes and all functional categories except frameshift insertions. This could indicate more positive selection on insertions than on deletions.

To date, a few TE insertions have been shown to have adaptive effects by adding specific regulatory regions, generating new transcripts, or inactivating genes (Daborn *et al.* 2002; Aminetzach *et al.* 2005; Chung *et al.* 2007; González *et al.* 2008, 2009; Schmidt *et al.* 2010; Magwire *et al.* 2011; Guio *et al.* 2014; Mateo *et al.* 2014). Others have been shown to affect different cellular processes such as the establishment of dosage compensation (Ellison and Bachtrog 2013), heterochromatin assembly (Sentmanat and Elgin 2012), or brain genomic heterogeneity (Perrat *et al.* 2013). Therefore, even though most TE insertions are present in the population at low frequencies because of purifying selection acting on them, others suggest some sort of selective advantage.

#### Idiosyncrasy of the *X* chromosome: the faster-*X* hypothesis:

The *X* chromosome exhibits a singular pattern of variation. Levels of polymorphism are similar (MW population) or lower (RAL population and other non-African populations; Grenier *et al.* 2015) than in autosomes, and polymorphism is weakly correlated with recombination rate. The *X* contains a higher percentage of gene regions undergoing both strongly deleterious and adaptive evolution, and a lower level of weak negative selection (Mackay *et al.* 2012). In contrast, divergence is greater for the *X* than for autosomes. The faster-*X* hypothesis (Charlesworth *et al.* 1987; Meisel and Connallon 2013) proposes that *X* chromosomes evolve more rapidly than autosomes because *X*-linked genes with favorable mutations that are recessive or partially recessive are more exposed to selection in hemizygous males than similar genes on autosomes. Prior to population genome studies, several attempts to test this hypothesis in *Drosophila* had led to opposite conclusions (Thornton *et al.* 2006; Connallon 2007; Baines *et al.* 2008). However, the different population genomics studies show unequivocally faster evolution of the *X* chromosome (Langley *et al.* 2012; Mackay *et al.* 2012; Campos *et al.* 2014). However, the higher exposure of mutations in hemizygous males to selection does not exclude recombination as another determining factor. The effective recombination rate is ∼1.8-fold greater on the *X* chromosome than autosomes (Barrón 2015). The increased selection on partially recessive alleles in hemizygous males together with the higher efficiency of selection due to the increased recombination in the *X* chromosome compared with the autosomes, may act synergistically to account for the faster *X* evolution.

### Geographic differentiation and demographic history

The demographic history of a population leaves a substantial footprint in the patterns of polymorphism and divergence, which can often confound the signatures of natural selection (Box 2). Modeling demography is thus of utmost importance, not only to trace the origin and expansion of a species, but also to make inferences about how natural selection and other evolutionary forces have shaped the genome.

We briefly review here the demographic history of *D. melanogaster*, a cosmopolitan species that originated from sub-Saharan Africa. The most recent studies about the demographic history of the ancestral Afrotropical population reveal a strong signature of a population bottleneck followed by population expansion about 60 KYA (Stephan and Li 2006; Singh *et al.* 2013). This expansion fostered the fixation of many beneficial mutations, leaving in the genome signatures of frequent selective sweeps (Box 2) (Stephan and Li 2006). The ancestral population colonized Europe and North America ∼19,000 and ∼200 years ago, respectively (Duchen *et al.* 2013), also leaving some signatures of local adaptive substitutions (Stephan and Li 2006). Finally, Pool *et al.* (2012) found evidences of admixture in all African *D. melanogaster* populations, with the fraction of introgression of cosmopolitan alleles into African populations ranging from <1 to >80% (Lack *et al.* 2015). This introgressed fraction of the genome has altered the patterns of genomic diversity irreversibly, *e.g.*, creating tracks of long-range LD and reducing population differentiation, and thus admixed DNA should be filtered from downstream population genomics analyses (Pool *et al.* 2012). Grenier *et al.* (2015) provides a reference collection of 84 strains of *D. melanogaster* from five continents.

Spatially and temporally varying selection also leaves complex signatures in the genome that may confound those left by demography, and thus may complicate further the interpretation of genetic variation data. Numerous examples of clinal variation have been published in *Drosophila*, including latitudinal, longitudinal, and altitudinal variation (Flatt 2016). Recently, Machado *et al.* (2016) examined the selective and demographic contributions to latitudinal variation through the largest comparative genomic study to date, using 382 complete individual genomes of *D. melanogaster* and *D. simulans*, finding more stable clinal variation in the former, and reporting a significant fraction of clinal genes that are shared between these species. Examples of cyclic changes in allele frequencies following the seasonal cycle have also been reported (Behrman *et al.* 2015). As a whole, even though we only briefly review the impact of geographic differentiation and demographic history on genomic variation, the reader can grasp the difficulties that these factors add to the interpretation of genetic variation in populations, and that distilling adaptive signal from demographic noise is a complex, laborious task (Flatt 2016).

## Determinants of Patterns of Genome Variation

### Recombination and linked selection

The first robust observation from population genomics studies is that local recombination rate affects the patterning of all types of variants (*e.g.*, SNP, indels, TE) along the genome, showing a positive correlation with the polymorphism level for every analyzed variant (Begun and Aquadro 1992; Mackay *et al.* 2012). This constitutes one of the most universal empirical observations of genome-wide population genetic analyses to date (Fay 2011; Smukowski and Noor 2011; for a contrasting view see Cutter and Payseur 2013). Mutation associated with recombination can be excluded as the cause of this correlation, at least in *Drosophila*, given the lack of correlation between recombination and divergence for SNPs and indels (Begun and Aquadro 1992; Mackay *et al.* 2012). Recombination itself, rather than any other factor, seems to be the main process determining the pattern of nucleotide diversity along the genome. Evolutionary models of recurrent linked selection, such as hitchhiking and BGS, predict a positive correlation between recombination and polymorphism for all variants (Berry *et al.* 1991; Begun and Aquadro 1992; Charlesworth *et al.* 1993; Huang *et al.* 2014). Thus, recombination rate via recurrent linked selection seems the likely explanation for the observed clustering of variants (Huang *et al.* 2014). This evidence can be interpreted as the vindication of the selective hitchhiking hypothesis *vs.* the (nearly) neutral hypothesis (Hahn 2008), such that the positive correlation between polymorphism and recombination reflects the footprint of natural selection in the genome. The degree to which linked selective sweeps reduce genetic diversity depends primarily on the rate of sweeps per genetic map length (Weissman and Barton 2012). This prediction has been corroborated in *Drosophila*: diversity increases with recombination rate and decreases with the density of functional sites (Begun *et al.* 2007; Shapiro *et al.* 2007). However, *Drosophila* is the most striking example of genetic draft. Because the *Drosophila* taxa has a large *N*_e_ compared with humans and other organisms studied to date, the evidence for adaptive selection in other species is not as prevalent and in some cases, as in humans, BGS seems to be the better explanation for the correlation between recombination and polymorphism (reviewed by Cutter and Payseur 2013).

### Pervasive selection and the HRi

**Figure 5:**
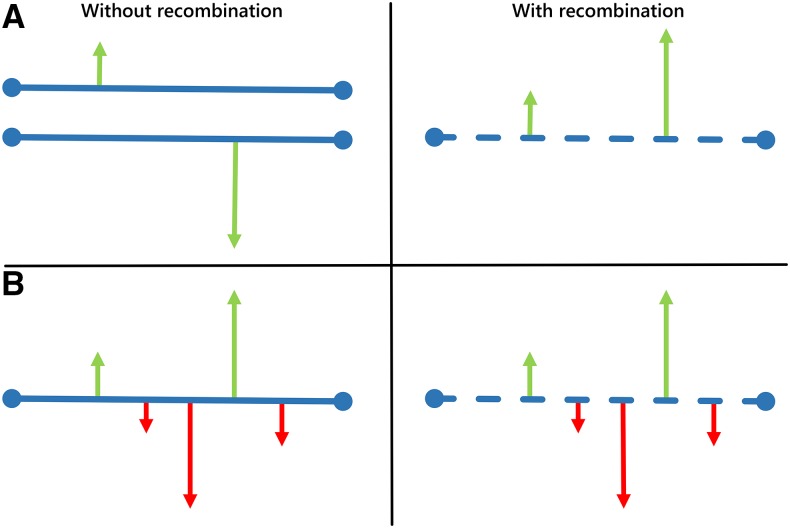
Representation of the cost of linkage on selected sites, or HRi. Arrows indicate adaptive (green) and deleterious (red) mutations, while their length indicates the intensity of selection. (A) When two or more adaptive mutations occur in separate haplotypes without recombination (left), only one of them can be fixed in the population and thus mutations compete for their fixation. However, when recombination is sufficiently high (right), the two haplotypes can exchange alleles and generate a new haplotype that carries both adaptive mutations and can be fixed. (B) In the presence of both adaptive and deleterious mutations without recombination (left), all alleles compete; as a result, deleterious alleles may be dragged to fixation if the intensity of selection favoring a nearby adaptive mutation is high, or adaptive alleles may be lost if the joint strength of negative selection is higher. With recombination (right), deleterious alleles can be removed and adaptive alleles can be fixed without interfering with each other. Adapted from Barrón (2015).

The second main observation from population genomics analyses is that adaptive and purifying selection is pervasive in the genomes of most studied species, especially in species such as *Drosophila* with a high *N*_e_. Deleterious mutations arise continuously, and genomes are populated by large numbers of segregating sites undergoing weak deleterious selection. Adaptive selection, as measured by the relative excess of divergence with respect to polymorphism by MK-like tests, is also ubiquitous. One implication of the large number of selected variants is that at any time there are genetic variants in LD simultaneously selected in the genome. These variants interfere with each other, inducing a cost of linkage known as the HRi (Hill and Robertson 1966). HRi is the evolutionary consequence of selection acting simultaneously among two or more cosegregating sites in finite populations, where the rate of adaptive (deleterious) fixation decreases (increases) as recombination decreases. Two scenarios exemplify HRi (Figure 5): (i) Two or more independent adaptive (+) mutations appearing in separated low-recombining haplotypes compete against each other for fixation, lowering the average rate of adaptive fixation; and (ii) deleterious (−) and adaptive variants coexist in a low-recombinant genome block. In this setting, some − variants are dragged to fixation by linked + variants, while the fixation rate of + variants is decreased due to the reduced efficacy of selection caused by linked − variants. HRi is predicted to be stronger in regions with lower recombination, a larger number of selected sites, and more intense selection (Comeron *et al.* 2008; Messer and Petrov 2013). If the cost of linkage is real, the number of selected variants undergoing HRi will increase as the recombination rate decreases. Conversely, regions with higher recombination rates will exhibit higher rates of adaptive fixation. Previous analyses of rates of protein evolution between *Drosophila* species showed that genes located in genomic regions with strongly reduced recombination have an excess of fixed deleterious mutations and a deficit of fixed advantageous mutations compared to highly recombining genomic regions (Takano 1998; Comeron and Kreitman 2000; Betancourt and Presgraves 2002; Zhang and Parsch 2005; Haddrill *et al.* 2007), supporting the role of HRi across genomes. The population genomics studies confirm and reinforce the importance of HRi (Langley *et al.* 2012; Mackay *et al.* 2012; Campos *et al.* 2014; Castellano *et al.* 2016). HRi can be caused either by selective sweeps of positively selected alleles or by BGS against deleterious mutations, but hitchhiking is not a sufficient condition for HRi. A sweep on a single selective target dragging linked neutral variants will reduce the level of polymorphism of affected regions, but it does not alter the adaptation rate of the region (Birky and Walsh 1988). HRi requires simultaneous targets of selection in LD.

### Quantifying the adaptive potential of a genome

If the HRi is common, a central question is its magnitude. How much does HRi limit the molecular adaptation of a genome? While different studies demonstrate the existence of HRi (reviewed by Comeron *et al.* 2008), it is not obvious *a priori* how to measure its amount in the whole genome. The chromosome length affected by HRi depends on the recombination rate and the distribution of linked fitness variation along the chromosome. The empirical correlation found between recombination and polymorphism is considered linear along the interval of recombination values (Smukowski and Noor 2011), and little attention has been paid to nonlinear relationships. Mackay *et al.* (2012) found a threshold value of recombination rate of ∼2 cM/Mb, above which recombination and nucleotide diversity become uncorrelated. What is the meaning of this threshold for recombination rate? For a given genome distribution of linked fitness variation, it can be hypothesized that there exists an optimal baseline value of recombination (*r*_opt_) above which any detectable HRi vanishes. Perhaps the recombination threshold value found by Mackay *et al.* (2012) represents *r*_opt_ for this species?

**Figure 6:**
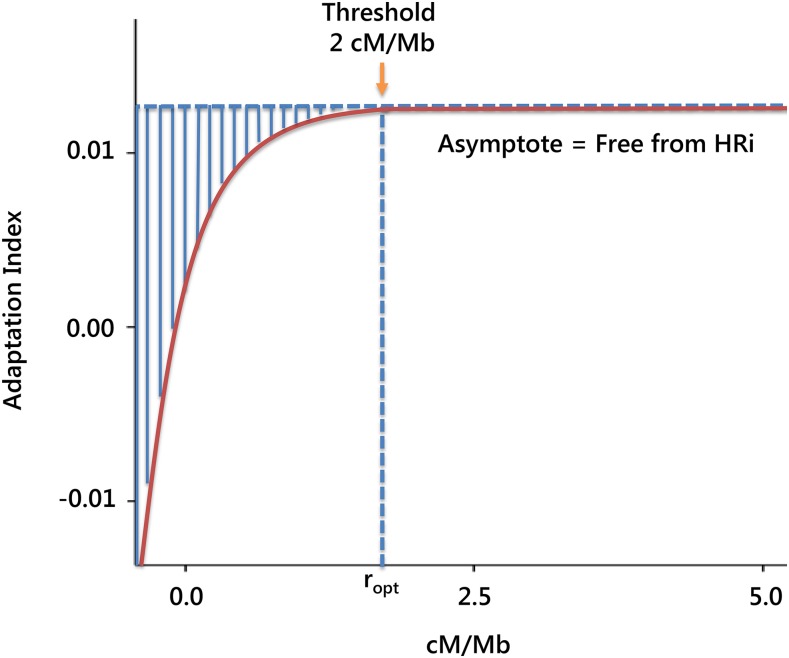
Relationship between recombination and adaptation in the *D. melanogaster* genome. The adaptation rate of a genomic region increases with the recombination rate until a threshold value of recombination (∼2 cM/Mb) in which adaptation rate reaches an asymptote. The shaded area represents the reduction of adaptive rate due to the cost of genome linkage, whose value has been estimated for the first time at ∼27% in a North American population of *D. melanogaster*. *r*_opt_ is the optimal baseline value of recombination above which any detectable HRi vanishes (see text for details). Adaptation index: *K_a+_*, rate of adaptive nonsynonymous substitution. Negative values mean fixation of deleterious mutations. Data from Castellano *et al.* (2016).

Castellano *et al.* (2016) measured the genomic impact of HRi by analyzing 6141 autosomal protein coding genes from the DGRP genome data. The rate of adaptive evolution (α) was calculated for this gene set using a derivative of the MK test (Eyre-Walker and Keightley 2009) which takes slightly deleterious mutations into account. When adaptation values were correlated with the high-resolution recombination estimates of Comeron *et al.* (2012), a clear positive correlation between recombination and adaptation was found. The surprise came when the initially observed linear relationship between recombination and adaptation converged to an asymptotic threshold in recombination values ∼2 cM/Mb, the same recombination threshold found by Mackay *et al.* (2012). This asymptote seems to indicate that the cost of linkage (the HRi) disappears for a given recombination value, above which the selected mutations behave as if they were freely segregating. In other words, an infinite recombination rate would not increase the genome adaptive rate of a region more than a recombination value of 2 cM/Mb (the estimated recombination threshold). The asymptote can then be interpreted as the *r*_opt_ for the adaptation rate of a genome, and its value informs about the background genome adaptation rate in the absence of linkage cost (Figure 6).

The determination of *r*_opt_ makes the estimation of the cost of HRi of a genome feasible. By comparing the average α value for genes residing in recombination regions ≥*r*_opt_ with the average α for the whole genome, the genome-wide impact of HRi on the adaptation rate can be quantified (shaded region in Figure 6). Castellano *et al.* (2016) estimate that HRi reduces the evolutionary adaptation rate of the *D. melanogaster* genome by 27%. Interestingly, genes with low mutation rates embedded in gene poor regions lose ∼17% of their adaptive substitutions, while genes with high mutation rates embedded in gene-rich regions lose ∼60% (Castellano *et al.* 2016). This does not necessarily mean that the HRi disappears above the *r*_opt_ value. Nearby mutations will probably experience HRi (Comeron and Guthrie 2005), but the bulk of selected mutations undergoing HRi is not large enough to affect α.

*r*_opt_ and the HRi load (*L*_HRi_) are two new parameters defining the adaptive potential of a genome that can be estimated with population genomics data (Table 1). Both parameters inform about the limits to adaptation imposed by linked selection and can be viewed as summarizing the historical interplay of population genetics forces acting on a genome. These two parameters should join the arsenal of parameters to estimate in future population genomic analyses. More estimates of these parameters in different populations and species are needed to understand the prevalence of HRi and to assess the importance of the different factors underlying the disparity in both linked selection and HRi among species.

## Population Genomics Challenges

### Baseline models of genome variation

If recurrent linked selection occurs in the genome, the nearly neutral theory is no longer the appropriate null model for the genome. A nearly neutral framework to analyze genome data would distort the interpretation of variation patterns (Hahn 2008). Given that slightly deleterious mutations populate genomes, BGS should be taken into account as a null model of molecular evolution (Lohmueller *et al.* 2011). Comeron (2014) has generated a first high-resolution landscape of variation across the *D. melanogaster* genome under a BGS scenario independent of polymorphism data. Simple models of purifying selection with the available annotations of recombination rates and gene distribution across the genome were integrated to obtain a baseline of genome variation predicted by the constant input and removal of slightly deleterious mutations. The results showed that ∼70% of the observed variation in diversity across the autosomes can be explained by BGS alone. BGS predictions can then be used as a baseline to infer additional types of selection and demographic events. In another study, Corbett-Detig *et al.* (2015) developed an explicit model combining BGS and hitchhiking and incorporating polymorphism, recombination rate, and density of functional elements in the genome to assess the impact of selection on neutral variation. Future population genome analyses should incorporate routinely realistic baseline models which allow performing more powerful, knowledge-based, population genomics tests.

### The HRi block as the unit of selection

The core theory of population genetics is built on freely segregating sites (Crow and Kimura 1970; Charlesworth and Charlesworth 2010). Consider a new mutation appearing in a population: Population genetics theory says that the probability of fixation $u\left(N_{e},s\right)$ is a single function of *N*_e_ and *s* (Kimura 1983, 1957) (see section *The distribution of fitness effects*). As shown above, |*N*_e_*s*| ≤ 1 defines the domain of the neutral realm *vs.* the selective one. But if HRi is common in genomes, then the unit of selection is no longer a freely segregating site, but a genome block formed by several targets of selection in joint LD. That is, the unit of genome selection is an HRi block whose length summarizes the historical interplay of mutation, selection, genetic drift, target density, and recombination acting on each genome region (Barrón 2015). Some consequences of the HRi can be accounted for by a reduction in the *N*_e_ of the affected region. However, if fitness interaction on selected sites is common, scaling *N*_e_ fails to capture the dynamic complexity of interacting sites. The probability of fixation of a selected mutation within an HRi block cannot be predicted without considering the fitness and LD relationships of the other selected variants. Trying to wrap the complexity of HRi into an effective parameter, such as *N*_e_, can introduce massive errors into the estimation of key population genetic parameters (Messer and Petrov 2013). The population dynamics of two or more selected sites in joint LD is extremely complex with no obvious solution and limited current knowledge (Charlesworth *et al.* 2009; Barton 2010; Neher 2013). New analytics need to be developed to take into account the complexities of linkage and HRi effects; forward simulation methods seem to be appropriate (Hernandez 2008; Messer and Petrov 2013).

### Positive selection and adaptation

From 30 to 50% of fixed nonsynonymous mutations in *D. melanogaster* are caused by positive selection (Eyre-Walker 2006; Mackay *et al.* 2012). What is the adaptive significance of this amount of positive selection? Positive selection and adaptive selection are typically considered to be synonymous terms. In genomic regions under HRi, weak deleterious mutations will be repeatedly fixed in the genome, increasing the opportunity for compensatory mutations that restores the harmful effect of the previously fixed deleterious mutations (Kimura 1985). It could be the case that many variants fixed by positive selection are such compensatory mutations. These mutations cannot be considered adaptation in a strong evolutionary sense, because adaptation implies an innovative new feature of an organism, while a compensatory change restores a previous trait to its normal function. It is a beneficial change but not an adaptation. Mustonen and Lässig (2009, 2010) proposed a new conceptual framework to distinguish positive selection from adaptation. Deleterious and beneficial mutations occur within the context of static DFE or selective equilibrium. An adaptation, however, is defined as a nonequilibrium response to changes in selection that implies a surplus of beneficial over deleterious changes reflected in a time-dependent fitness landscape. If most of the estimated positive selection is due to compensatory substitution, then this evidence says little about adaptation. If adaptation is a multilevel process that concerts phenotypic-genotypic changes by adjusting multilevel constraints, population genomics data have to be integrated with other phenotypic multi-omics data to obtain a complete picture of how adaptation occurs (see *The future: Toward a Population -Omics Synthesis*).

### Nonequilibrium theory

Theoretical predictions and tests for selection applied to genetic variation data are generally based on the assumption that populations are at a demographic equilibrium. However, demographic fluctuations must occur in every natural population. Most populations of model species studied have experienced recent changes in population sizes, recombination, and other genome features (see section *Geographic differentiation and demographic history*). If the equilibrium assumption is violated, estimates of both positive and deleterious selection can be seriously biased (Jensen *et al.* 2005; Pool *et al.* 2012; Singh *et al.* 2013). For example, in a population that has suffered a bottleneck followed by an exponential growth, deleterious mutations reach equilibrium frequencies more quickly than neutral mutations, which can be interpreted as an excess of segregating deleterious variants in the population when applying a test for selection (Brandvain and Wright 2016). The nonequilibrium world should be more widely developed to include more realistic models that explicitly incorporate nonequilibrium dynamics, selection tests robust to departures from equilibrium, and use simulation of molecular data (Carvajal-Rodríguez 2008; Arenas 2012).

### *N*_e_ *vs.* *N_c_*

*N*_e_ is a parameter that captures long-term population dynamics. *N_e_* is usually estimated from the levels of standing variation, which is very sensitive to past bottleneck events. Because the number of new beneficial mutations entering a population at a given moment depends on the census population size, focusing on *N*_e_ to assess the adaptive potential of a species can be seriously misleading. Consider the key insecticide resistance locus *Ace* in *D. melanogaster*. It evolved quickly, repeatedly incorporating resistance alleles within individual populations (Karasov *et al.* 2010). The inferred number of reproducing flies required to account for the repeated convergent mutations is ∼10^9^, >100-fold larger than the estimated *N*_e_. This observation has two important consequences: (1) adaptation in *Drosophila* may not be limited by waiting for beneficial mutations at single sites; and (2) multiple convergent mutations or standing variants can be fixed, leaving a weaker signature on the pattern of variation, the so-called soft sweep (Karasov *et al.* 2010). In contrast, the standard sweeps of positive directional selection, also called hard sweeps, assume a mutation-limited scenario where a single beneficial mutation is selected in each iterative sweep, leaving a stronger footprint on the pattern of variation. In *D. simulans*, it has been estimated that ∼13% of replacement-site substitutions were fixed by hard selection *vs.* ∼90% fixed through either hard or soft sweeps (Sattath *et al.* 2011). In *D. melanogaster*, whole-genome data has been used to demonstrate that elevated long-range LD and signatures of soft sweeps are present in different populations of this species (Garud *et al.* 2015; Garud and Petrov 2016). The relative incidence of hard *vs.* soft selection is an unsolved problem. The impact of the disparity between effective and census (actual) population sizes has to be considered for other parameters or combination of parameters based on *N*_e_; for example, the historical recombination parameters, ρ = 4*N*_e_*r*, or the vulnerability to hitchhiking effect index, ρ/4*N*_e_*µ* (Lynch 2007).

## The Future: Toward a Population -Omics Synthesis

Population genomics studies to date have been limited to the genotypic space: the description of genome variation patterns of individuals of different populations and species, while trying to discern the relative importance of the evolutionary forces modeling these patterns. However, natural selection acts primarily on the phenotype, while leaving its footprint on the genotype. The genomic dimension, albeit necessary, is not sufficient to account for a complete picture, retrospective and prospective (He and Liu 2016), of organismal adaptation (Lewontin 2000).

Recent advances in NGS technologies have boosted the breadth of available -omics data, from the genomic level to epigenomic, transcriptomic, proteomic, or metabolomic data. These different -omics layers, which in contrast to the genomic sequence vary during the lifetime of an individual and in different parts of the body, represent intermediate phenotypes between the genomic space and the final organismal phenotype on which natural selection operates (Civelek and Lusis 2014). While a single -omics layer can only provide limited insight into how different evolutionary forces have shaped this particular -omics layer through their action on the phenotype; the integration of multiple -omics layers across time and space (*e.g.*, measuring the action of natural selection in genes specifically expressed in different organs or across development), and the study of their causal relationships, promises to provide a systemic view of the causes and consequences of evolutionary and functional effects of genomic variation, as well as further our understanding of important biological processes underlying complex-trait architecture (Ayroles *et al.* 2009; Massouras *et al.* 2012; Ritchie *et al.* 2015).

The genome sequences and phenotype data of the DGRP (Mackay *et al.* 2012; Huang *et al.* 2014); together with the high-resolution QTL mapping data of the *Drosophila* Synthetic Population Resource (King *et al.* 2012; Long *et al.* 2014); and the multi-omics data from the *Drosophila* model organism Encyclopedia of DNA elements (modENCODE) project, including mapped transcripts, histone modifications, chromosomal proteins, transcription factors, replication proteins and intermediates, and nucleosome properties across a developmental time course and in multiple cell lines (Consortium *et al.* 2010); are gold mines of data that are irreversibly changing population genetics. In addition, “evolve and resequence” experiments analyze rapid phenotypic responses to laboratory selection, followed by NGS to identify the individual loci underlying adaptation (Kofler and Schlötterer 2014; Long *et al.* 2015). The description and integrative analysis of intra- and interpopulation genome-wide multi-omics data are now feasible and should soon provide a unified fitness–phenotype–genotype map on which to extend the population genetics theory toward a systemic evolutionary theory.

Population genetics is no longer an empirically insufficient science, but it is more than ever a research field where bioinformatics tools for data mining and management of large-scale data sets, statistical and evolutionary models, and advanced molecular techniques of massive generation of sequences are all integrated in an interdisciplinary endeavor. At the heart of the -omics momentum—and rephrasing Dobzhansky’s famous dictum (Lewontin 1991)—this brief journey over the golden anniversary of molecular population genetics leads us conclude that “The problematic of population genomics is the description and explanation of multi-omics variation within and between populations.” New and exciting challenges await the next 50 years!
